# Supplementary material for: Symmetry-protected hierarchy of anomalous multipole topological band gaps in nonsymmorphic metacrystals
Source: Nat Commun. 2020 Jan 3;11:65. doi: 10.1038/s41467-019-13861-4 (PMC6941978; doi:10.1038/s41467-019-13861-4)
Supplement: Supplementary file 1 — Supplementary Information [file 41467_2019_13861_MOESM1_ESM.pdf]

Supplementary Information - *Symmetry-protected anomalous multipole topological insulators in nonsymmorphic metacrystals,* Zhang *et al.*

Table of Content

Supplementary Note 1: Symmetry constraints on Wannier bands and nested Wannier bands 3
Supplementary Note 2: Effects of the glide symmetries on the band structure, Bloch states, parity and band topology 6
Supplementary Note 3: Numerical calculation of the Wannier bands and nested Wannier bands 9
Supplementary Note 4: Bulk-edge-corner correspondence in wallpaper *p*4*g* crystals 11
Supplementary Note 5: Validity of the 2D approximation 12
Supplementary Note 6: Topological transition for the quadrupole moment 13
Supplementary Note 7: Robustness of the quadrupole topological edge and corner states 19
Supplementary Note 8: Measurements of the quadrupole edge gap and the transmission spectra for various pump-probe configurations 23
Supplementary Note 9: Real-part of the acoustic field for the corner state from experiments 26
Supplementary Note 10: Calculated edge and corner states and topological transition in the second band gap with different widths of the air-channel 27
Supplementary Note 11: Topological transition for the first bulk band gap with different geometry parameters 28
Supplementary Note 12: Hamiltonian theory of the Dirac point and the first acoustic band gap 29
Supplementary Note 13: Topological index for the first band gap 32
Supplementary Note 14: Glide symmetry for the edges in Figure 5 and its physical consequence 33
Supplementary Note 15: Robustness of the gapless edge states in the first band gap. 34
Supplementary Note 16: The helical edge states and pseudospin-dependent edge state propagation 35
Supplementary References 36

Supplementary Note 1: Symmetry constraints on Wannier bands and nested Wannier bands

In this section, we present the symmetry constraints on the Wannier bands and the nested Wannier bands. We focus on the constraints imposed by the glide symmetries. Regarding the other symmetries, such as the inversion and C_4 rotation symmetries, their effects on the Wannier bands and nested Wannier bands have been analyzed in Supplementary references [1, 2].

Following Supplementary references [1, 2], the constraint of a symmetric operator D_g over the Wilson loop $\mathcal{W}_{\mathcal{C},\mathbf{k}}$ satisfies the following relation

$$B_{g,\mathbf{k}}\mathcal{W}_{\mathcal{C},\mathbf{k}}B_{g,\mathbf{k}}^{+} = \mathcal{W}_{D_g\mathcal{C},D_g\mathbf{k}}. \quad (1)$$

Here, in the Wilson loop, $\mathcal{W}_{\mathcal{C},\mathbf{k}}$, the first subscript $\mathcal{C}$ is the contour along which the Wilson loop is performed, and the second subscript $\mathbf{k}$ is the starting point of the Wilson loop. $B_{D_g\mathbf{k}}^{mn} =< u_{D_g\mathbf{k}}^{m}|g_{\mathbf{k}}|u_{\mathbf{k}}^{n} >$ is the unitary sewing matrix that connects the Bloch states at $\mathbf{k}$ with those at $D_g\mathbf{k}$, which have the same energy. $g_{\mathbf{k}}$ is the unitary matrix operator corresponding to the symmetry operator D_g, which transforms the Hamiltonian $h_{\mathbf{k}}$ as following

$$g_{\mathbf{k}}h_{\mathbf{k}}g_{\mathbf{k}}^{-1} = h_{D_g\mathbf{k}}. \quad (2)$$

We define the Wilson loops along the contour $\mathcal{C} = (k_x, k_y) \to (k_x + \frac{2\pi}{a}, k_y)$ with increasing (decreasing) k_x as $\mathcal{W}_{x,\mathbf{k}}$ ($\mathcal{W}_{-x,\mathbf{k}}$), where $\mathbf{k} = (k_x, k_y)$ is the starting point of the Wilson loop. Similarly, for the path $\mathcal{C} = (k_x, k_y) \to (k_x, k_y + \frac{2\pi}{a})$ with increasing (decreasing) k_y, we denote the Wilson loops as $\mathcal{W}_{y,\mathbf{k}}$ ($\mathcal{W}_{-y,\mathbf{k}}$). Supplementary Figure 1 shows how those Wilson loops transform under the glide reflections G_x and G_y. We notice that under these glide reflections, $G_x\mathbf{k} = G_x(k_x, k_y) = (-k_x, k_y)$, and $G_y\mathbf{k} = G_y(k_x, k_y) = (k_x, -k_y)$. Therefore, in the wavevector space, the effects of G_x are equivalent to that of the mirror reflection $M_x: (x, y) \to (-x, y)$ studied in Supplementary references [1, 2]. Similarly, the effects of G_y are equivalent to that of the mirror reflection $M_y: (x, y) \to (x, -y)$. Thus, we can safely inherit the theory from Supplementary references [1, 2] to derive the constraints on the (nested) Wannier bands imposed by the glide reflection symmetries. We shall not represent the mathematical details, but use rather intuitive and heuristic arguments to achieve this goal.

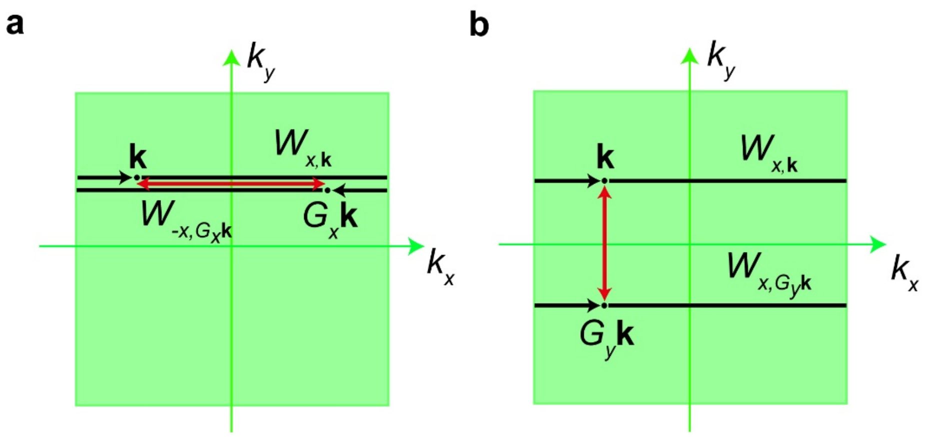

Supplementary Figure 1 | Relations between the Wilson loops along the k_x direction with a base point k after a, glide operation G_x and b, glide operation G_y.

a. Constraints on the Wannier bands due to G_x

The conclusion is that under the glide symmetry G_x, the eigenvalues of the Wilson loops along the k_x direction, $\mathcal{W}_{x,\mathbf{k}}$, are constrained to be 1, -1 or to come in complex-conjugated pairs $e^{\pm i2\pi\nu}$.

This is because $G_x\mathbf{k} = G_x(k_x, k_y) = (-k_x, k_y)$. The relation between the Wilson loops in Supplementary Equation (1) for this symmetry is

$$B_{G_x\mathbf{k}}\mathcal{W}_{x,\mathbf{k}}B^{+}_{G_x\mathbf{k}} = \mathcal{W}_{-x,\mathbf{k}} = \mathcal{W}^{+}_{x,G_x\mathbf{k}}, \tag{3}$$

where $B^{mn}_{G_x\mathbf{k}} =< u^{m}_{G_x\mathbf{k}}|g_{\mathbf{k}}|u^{n}_{\mathbf{k}} >$ is the unitary sewing matrix that connects states at $\mathbf{k}$ with those at $G_x\mathbf{k}$, which have the same energy, and $g_{\mathbf{k}}$ is the unitary matrix operator. Thus, the Wilson loop at $\mathbf{k}$ is equivalent (up to a unitary transformation) to the Hermitian conjugate of the Wilson loop at $G_x\mathbf{k}$. Since the eigenvalues of the Wilson loop $\mathcal{W}_{x,\mathbf{k}}$ are k_x independent, this directly yields

$$\left\{e^{i2\pi\nu_x^{j}(k_y)}\right\} = \left\{e^{-i2\pi\nu_x^{j'}(k_y)}\right\} \tag{4}$$

or

$$\nu_x^{j}(k_y) = -\nu_x^{j'}(k_y)\, mod\ 1 \tag{5}$$

The two indices, j and j', do not have to be the same. Thus, the Wannier centers are forced to 0 (centered in the unit cell), 0.5 (centered in-between unit cells), or to come in pairs $(-\nu, \nu)$ (which are equally displaced from the unit-cell center but at opposite sides).

b. Constraints on the Wannier bands due to G_y

Since $G_y\mathbf{k} = G_y(k_x, k_y) = (k_x, -k_y)$, the glide symmetry G_y connects two

Wilson loops along the k_x direction with opposite $-k_y$. Following the above procedure and analysis, one has

$$\left\{e^{i2\pi v_x^j(k_y)}\right\} = \left\{e^{i2\pi v_x^{j'}(-k_y)}\right\} \tag{6}$$

$$v_x^j(k_y) = v_x^{j'}(-k_y)\ mod\ 1 \tag{7}$$

c. Constraints on the nested Wannier bands due to G_x

Using Supplementary Equation (5) and keeping in mind that in the reciprocal space $G_x\mathbf{k} = G_x(k_x, k_y) = (-k_x, k_y)$, intuitively, the invariance of the nested Wilson loops under the glide symmetry G_x is written as

$$\{\exp[i2\pi v_y^{v_x,j}(k_x)]\} = \left\{\exp\left[i2\pi v_y^{-v_x,j'}(-k_x)\right]\right\} \tag{8}$$

or

$$v_y^{v_x,j}(k_x) = v_y^{-v_x,j'}(-k_x)\ mod\ 1, \tag{9}$$

where $j, j' \in 1 \ldots N_{v_x}$ label the eigenvalues, and N_{v_x} is the number of Wannier bands in the sector v_x. The Wannier-sector polarization can be written as

$$p_y^{v_x} = \frac{1}{N_x}\sum_{k_x}\sum_{j=1}^{N_{v_x}} b_j v_y^{v_x,j}(k_x)\ mod\ 1, \tag{10}$$

where $b_j = \pm 1$ represent different combinations (sectors) of the Wannier bands. Since here k_x is a dummy variable, the G_x symmetry implies that

$$p_y^{v_x} = p_y^{-v_x}\ mod\ 1. \tag{11}$$

d. Constraints on the nested Wannier bands due to G_y

Using Supplementary Equation (7) and keeping in mind that in the reciprocal space $G_y\mathbf{k} = (k_x, -k_y)$, intuitively, the invariance of the nested Wilson loops under the glide symmetry G_y should be

$$\{\exp[i2\pi v_y^{v_x,j}(k_x)]\} = \left\{\exp\left[-i2\pi v_y^{v_x,j'}(k_x)\right]\right\} \tag{12}$$

or

$$v_y^{v_x,j}(k_x) = -v_y^{v_x,j'}(k_x)\ mod\ 1. \tag{13}$$

Since here k_x is a dummy variable in Supplementary Equation (10), the G_y symmetry implies that

$$p_y^{v_x} = -p_y^{v_x}\ mod\ 1. \tag{14}$$

e. Quantization of the quadrupole moment

Summarizing the above constraints, we find that for even number of acoustic bands, there exist gapped Wannier bands that are symmetric around $\nu_x = 0, \frac{1}{2}$ lines, according to Supplementary Equation (5). Moreover, the Wannier bands are symmetric around $k_y = 0$, according to Supplementary Equation (7).

Putting the above constraints in Supplementary Equation (11) and (14) together, we find that,

$$p_y^{\nu_x} = 0, \frac{1}{2} \; mod \; 1, \tag{15}$$

for both Wannier sectors. Since the *x* and *y* directions are identical for the system with C_4 symmetry, we also have,

$$p_x^{\nu_y} = p_y^{\nu_x} = 0, \frac{1}{2} \; mod \; 1. \tag{16}$$

With above Wannier-section polarizations, the quantization of the quadrupole moment is deduced as in Supplementary reference [1,2],

$$q_{xy} = 2 p_x^{\nu_y} p_y^{\nu_x} = 0, \frac{1}{2} \; mod \; 1. \tag{17}$$

Therefore, the quadrupole moment is quantized by the glide symmetries.

Supplementary Note 2: Effects of the glide symmetries on the band structure, Bloch states, parity and band topology

In our sonic crystal (SC), the scatterers are arranged in a way to generate two orthogonal glide symmetries, $G_x := (x, y) \to (\frac{a}{2} - x, \frac{a}{2} + y)$ and $G_y := (x, y) \to (\frac{a}{2} + x, \frac{a}{2} - y)$. The first acoustic band evolves from the $\omega \to 0$ and $|\mathbf{k}| \to 0$ limit where it becomes plane-wave alike to finite frequencies. In this way, the first two acoustic bands are associated with the same representation of G_y with the eigenvalue $g_y = e^{ik_x a/2}$, and are connected by the glide symmetry, as shown in Supplementary Figure 2. This can simply be understood as that after a $\frac{2\pi}{a}$ interval, the first acoustic band evolves into the second acoustic band.

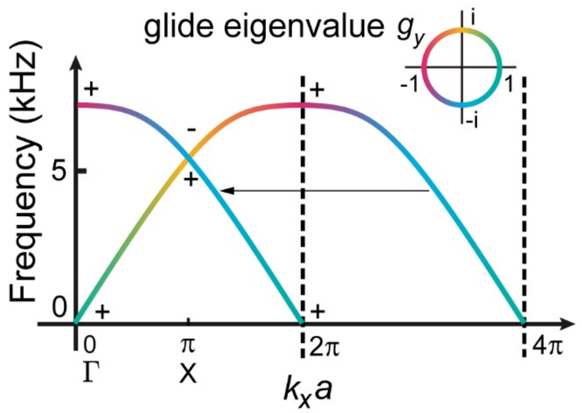

Supplementary Figure 2 | Evolution of the glide eigenvalue g_y (shown by color; the color map is at the upper-right) of the first two acoustic bands along the ΓX direction. The symbols $+/-$ represent even/odd parity, respectively, at the Γ and X points. The black arrow indicates folding the dispersion and the glide eigenvalue into the first Brillouin zone.

The glide symmetries result in double degeneracy on the XM and YM lines. This double degeneracy can be understood via constructing the anti-unitary operators, $\Theta_j = G_j * T$ $(j = x, y)$, where T is the time-reversal operator. Since $\Theta_y^2 \psi_{n,\mathbf{k}} = e^{ik_x a}\psi_{n,\mathbf{k}}$ at $k_x a = \pi$, $\Theta_y^2 = -1$ leads to Kramers-like double degeneracy. Such degeneracy also appears for $k_y a = \pi$ because of $\Theta_x^2 = -1$. Therefore, all acoustic bands are doubly degenerate on the Brillouin zone boundary (i.e., the XM and YM lines).

Moreover, there is a relation between the parity operator $\mathcal{P} \coloneqq (x, y) \to (-x, -y)$ and the glide operators, which is $\mathcal{P} = \mathcal{T}_{-y} * G_x * G_y$. Here $\mathcal{T}_{-y}$ is the translation along the $-y$ direction of a lattice constant (note that the two glide symmetries do not commutate with each other, i.e., $G_x G_y \neq G_y G_x$; one has, instead, a special relation, $\mathcal{T}_{-y} G_x G_y = \mathcal{T}_{-x} G_y G_x$). Considering the evolution of the glide operators along the ΓX and ΓY directions, as schematically shown in Supplementary Figure 3, the G_y operator has 1D representation along the ΓX direction, while the G_x operator has 1D representation along the ΓY direction. Since after a $\frac{2\pi}{a}$ interval the first acoustic band evolves into the second acoustic band, the parities of the two acoustic bands at the Γ point are connected with each other through $\frac{\mathcal{P}_2(\Gamma)}{\mathcal{P}_1(\Gamma)} = \frac{g'_x g'_y}{g_x g_y}$, where g_x, g_y and g'_x, g'_y are the glide eigenvalues at the Γ point for the first and the second acoustic bands, respectively. Here, $\mathcal{P}_2(\Gamma)$ and $\mathcal{P}_1(\Gamma)$ are used to denote the parities of the second and the first acoustic bands at the Γ point. We notice that the Γ point at the second band can be regarded as equivalent to the $(2\pi, 0)$ or the $(0, 2\pi)$ point (see Supplementary

Figure 3 for illustration). Inserting $g_y = \pm e^{ik_x a/2}$ and $g_x = \pm e^{ik_y a/2}$, one finds that $g_x' = -g_x$ and $g_y' = -g_y$. Therefore, the parities of the first and the second acoustic bands at the Γ point are identical, i.e., $\mathcal{P}_2(\Gamma) = \mathcal{P}_1(\Gamma)$. The above proof holds for each pair of bands that are degenerate at the XM line (as ensured by the glide reflection symmetries), if there is no degeneracy at the Γ point between the bands belong to different pairs.

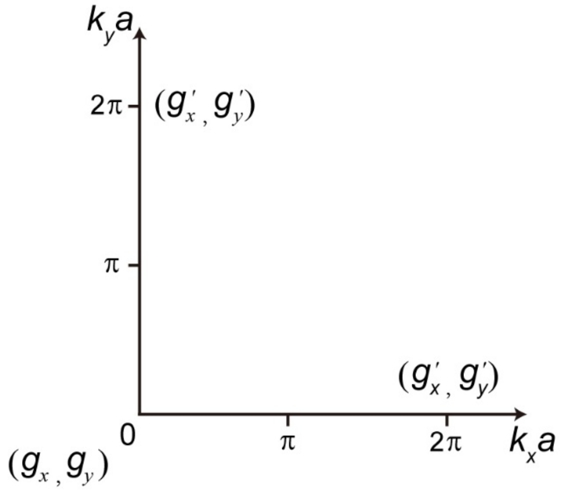

Supplementary Figure 3 | Illustration of the evolution of the glide operators and the parity operators in the Brillouin zone.

We now prove that the doubly degenerate acoustic states at the X (Y) point are of opposite parities. For this purpose, we study the commutation relation between the operators $\mathcal{P}$ and Θ_y. We find that $\mathcal{P}\Theta_y = -\Theta_y\mathcal{P}$ at the X point, because at the X point one has $\mathcal{P} = G_x * G_y = -G_y * G_x$. Since the two degenerate states at the X point can be written as $\psi_{n,\mathbf{k}}$ and $\Theta_y\psi_{n,\mathbf{k}}$, we find that $\mathcal{P}\Theta_y\psi_{n,\mathbf{k}} = -\Theta_y\mathcal{P}\psi_{n,\mathbf{k}}$ and thus the two states have opposite parities. The same property holds for the Y point, due to the C_4 symmetry.

Putting the above analyses together, whenever there is a pair of such acoustic bands that are connected to each other by the glide operators, they have the same parity at the Γ point, whereas they have the opposite parity at the X (Y) point. The topological dipole moment can be directly obtained from these parity properties as $\mathbf{p} = \left(\frac{1}{2}, \frac{1}{2}\right)$, using the theoretical results in Supplementary reference [3].

The degeneracy at the Γ point is determined by the C_4 point group. For odd-parity states, they are doubly degenerate. In contrast, the even-parity states are non-degenerate at the Γ point. In our case, the first two acoustic bands are of even parity at

the Γ point, while the third and the fourth acoustic bands are of odd parity at the Γ point.

Supplementary Note 3: Numerical calculation of the Wannier bands and nested Wannier bands

To obtain the Wannier bands and nested Wannier bands, we calculate the Wilson loop operator and the nested Wilson loop operator for the lowest four acoustic bands. The phase spectra of these two unitary operators (matrices) give, respectively, the Wannier bands and the nested Wannier bands. For such a purpose, we first calculate the wavefunctions of the acoustic Bloch states in the whole Brillouin zone using the finite-element numerical software COMSOL Multiphysics. We extract the numerical data of these wavefunctions and save them into a matrix form using 200×200 grids in the unit-cell. The Brillouin zone is discretized into 30×30 k-points, of which k_x is split into an array k_{x_1}, k_{x_2}, k_{x_3}, ... , k_{x_N} for each k_y, with N=30. k_{x_1} and k_{y_1} are indentical to k_{x_N} and k_{y_N}, respectively. To fix a periodic gauge, we replace the wavefunctions $\psi_n(k_{x_N},\mathbf{r})$ and $\psi_n\left(k_{y_N},\mathbf{r}\right)$ with $\psi_n(k_{x_1},\mathbf{r})$ and $\psi_n\left(k_{y_1},\mathbf{r}\right)$, respectively, where n = 1, 2, 3, 4 is the band index and $\boldsymbol{r}$ is the real-space coordinate. The periodic part of the Bloch wavefunctions are obtained through $u_n(\mathbf{k},\mathbf{r}) = e^{-i\mathbf{r}}\psi_n(\mathbf{k},\mathbf{r})$. Once all the $u_n(\mathbf{k},\mathbf{r})$ are prepared, the Wilson loop operators can be calculated directly. We consider the Wilson loop operators in the x direction, $\mathcal{W}_{x,\mathbf{k}}$, where $\mathbf{k}=(k_x,k_y)$ is the starting point of the loop. By defining $\left[F_{x,\mathbf{k}}\right]^{mn} = \langle u^m_{\mathbf{k}+\Delta k_x}|u^n_{\mathbf{k}}\rangle$, for $\Delta k_x = 2\pi/29$, $m,n=1,2,3,4$, the Wilson loop is obtained as $\mathcal{W}_{x,\mathbf{k}} = \prod_{i=0}^{28} F_{x,\mathbf{k}+i\Delta k_x}$. We then diagonalize the Wilson loop operator $\mathcal{W}_{x,\mathbf{k}}|\nu^j_{x,\mathbf{k}}\rangle = e^{2\pi i\nu^j_x(k_y)}|\nu^j_{x,\mathbf{k}}\rangle$, where j = 1, 2, 3, 4 is the Wannier band index, and $|\nu^j_{x,\mathbf{k}}\rangle$ is the eigenvector which depends on both the loop and the starting point of the loop. The phase ν^j_x, which depends only on the Wilson loop, is the Wannier center of the acoustic Bloch waves. As k_y goes from k_{y_1} to k_{y_N}, the evolution of ν^j_x forms the Wannier bands, which are shown in Fig. 1c in the main text.

When the Wannier bands are gapped, we can calculate the nested Wannier bands. The Wannier bands are split into two sectors, $\nu^-_x = \{\nu^j_x\,|j=1,3\}$ and $\nu^+_x =$

$\{\nu_x^j\,|j=2,4\}$, which separate the eigenstates of the first four Bloch bands into two Wannier band subspaces: $|w_{x,\mathbf{k}}^{\pm}\rangle=\sum_n|u_{\mathbf{k}}^n\rangle\,[\nu_{x,\mathbf{k}}^{\pm}]^n$, where the superscripts $\pm$ denote the Wannier sectors, $n=1,2,3,4$ is the Bloch band index for $|u_{\mathbf{k}}\rangle$, and $[\nu_{x,\mathbf{k}}^{\pm}]^n$ is the nth component of the Wannier eigenvector $|\nu_{x,\mathbf{k}}^{\pm}\rangle$.

Following the Supplementary references [1, 2], we define $[F_{y,\mathbf{k}}^{\pm}]^{mn}=\langle w_{x,\mathbf{k}+\Delta k_y}^{\pm,m}|w_{x,\mathbf{k}}^{\pm,n}\rangle$, for $\Delta k_y=2\pi/29$. Here, $m,n=1,3$ for the superscript $-$, while $m,n=2,4$ for the superscript $+$. The nested Wilson loops along the k_y direction are defined as $\widetilde{\mathcal{W}}_{y,\mathbf{k}}^{\pm}=\prod_{i=0}^{28}F_{y,\mathbf{k}+i\Delta k_y}^{\pm}$. We then diagonalize the nested Wilson loop operator. For each k_x, there are two eigenvalues, $e^{2\pi i p_y^{\nu_x^j}(k_x)}$ and $e^{2\pi i p_y^{\nu_x^{j'}}(k_x)}$, where j and j' respectively equal to 1 and 3 (2 and 4) for the Wannier sector ν_x^- (ν_x^+). The corresponding bulk-induced topological polarizations are then determined as $p_y^{\nu_x^{\pm}}(k_x)=p_y^{\nu_x^j}(k_x)+p_y^{\nu_x^{j'}}(k_x)$, which are quantized to ±0.5 as shown in Supplementary Figure 4. Importantly, it is also noted from Supplementary Figure 4 that the results for the nested Wannier bands in the combination "1-2" is the same as those for "1+3". And that for "4-3" is the same as those for "2+4". These properties can be easily understood from the fact that the Wannier bands and nested Wannier bands are symmetric around the $\nu_x=0$ and the $p_y^{\nu_x}=0$ lines: "3" is equivalent to "-2", and hence "2" is equivalent to "-3". The results in Supplementary Figure 4 indicate that the bulk-induced edge polarizations $p_y^{\nu_x^{\pm}}$ and the quadrupole moment is quantized in these Wannier sectors, which agrees with the analytical proof in Note 1 of the Supplementary Information.

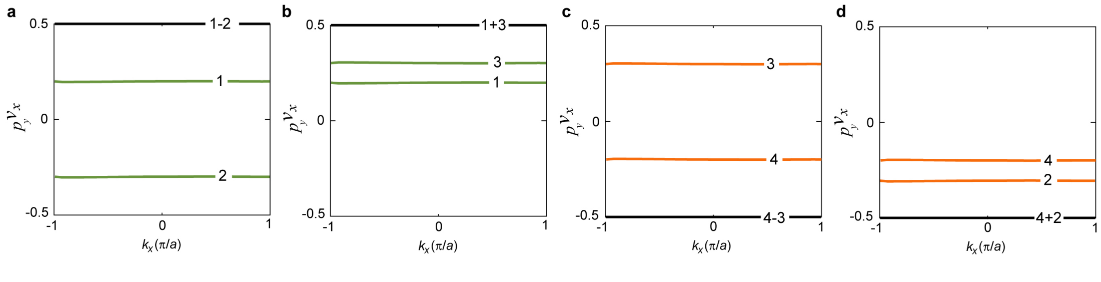

Supplementary Figure 4 | Nested Wannier bands calculated for the relative Wannier sectors. **a,** The nested Wannier bands for the ν_x^- sector ("1-2"). This is equivalent to a different combination ("1+3") as shown in **b. c,** The nested Wannier bands for the ν_x^+ sector ("4-3"). This is equivalent to

a different combination ("4+2") as shown in **d.**

Supplementary Note 4: Bulk-edge-corner correspondence in wallpaper *p*4*g* crystals

In this section, we elaborate on the bulk-edge and bulk-corner correspondences in the nonsymmorphic crystals that do not have mirror symmetries on the edges. The bulk-edge correspondence originates from the fact that the gapped Wannier bands are adiabatically connected to the gapped edge states. This bulk-edge connection is the same as that in Supplementary references [1, 2]. The slight difference here is that the gapped Wannier bands emerge in the difference sector. Although the sum sector ("1+2" or "3+4") has gapless Wannier bands which correspond to quantized dipole moments, the dipole moments have no consequence on the edges since the total dipole polarization of the four Wannier bands vanishes (both the "1+2" and "3+4" sectors have a dipole moment $\mathbf{p} = \left(\frac{1}{2}, \frac{1}{2}\right)$ and these two dipole moments cancel with each other given that the dipole moment is a modulo 1 quantity in both x and y directions).

As inferred from the nested Wannier bands, the bulk-induced edge polarizations at different edges due to the quadrupole topology are schematically illustrated in Supplementary Figure 5a. Differing from Supplementary references [1, 2], here, due to the lack of mirror symmetries on the edges, there is a geometric contribution to the edge polarization (see Supplementary Figure 5b). This geometry-induced edge polarization, p_{geom}, is due to the fact that the center of the edge wavefunction (along the edge) is not at the center of the unit-cell. Specifically, we plot the 1D profile of the integrated acoustic pressure field, $\int dy\, |P_{\text{edge}}(x, y)|\text{^}2$, for the edge state along the x direction (at $k_x = \frac{\pi}{a}$), in Supplementary Figure 5b (above the green dashed line). It is seen that the acoustic field profile is asymmetric, because the edge does not have mirror symmetry. In accordance with the C_4 symmetry, we have the configurations of edge polarization shown in Supplementary Figure 5a. Specifically, one has

$$p_y^{v_x^-} = -p_y^{v_x^+} = -p_x^{v_y^+} = p_x^{v_y^-} = \frac{1}{2}. \quad (18)$$

Taking both the bulk quadrupole moment and the bulk-independent, geometry-induced edge polarization into consideration, the "fractional charge" at the upper-right corner is equal to

$$Q_{\text{corner}}^{++} = q_{xy} + \left(p_x^{v_y^+} + p_{\text{geom}}\right) + \left(p_y^{v_x^+} - p_{\text{geom}}\right) = q_{xy} = \frac{1}{2}. \quad (19)$$

It is seen that due to the C_4-symmetric nature of the geometry-induced edge

polarization, it does NOT contribute to the corner charge. Therefore, the corner charge pattern remains the same as that in the conventional quadrupole topological insulators.

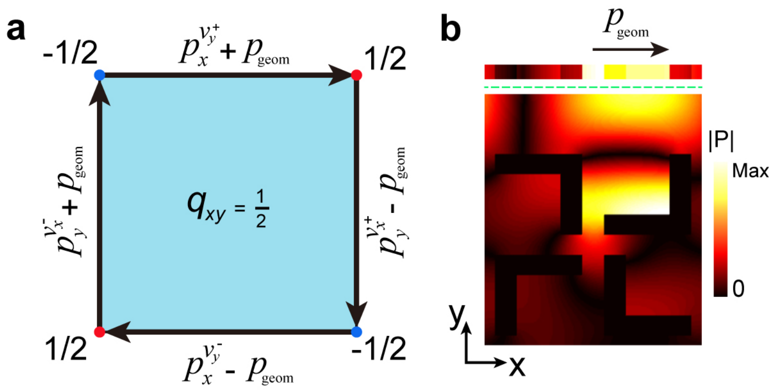

Supplementary Figure 5 | Schematic of a C_4-symmetric quadrupole insulator with no mirror symmetries. a, The bulk quadrupole q_{xy}, the bulk-induced edge polarizations and the geometry-induced edge polarizations. The arrows in **a** illustrate the C_4-symmetric configuration of the geometry-induced edge polarization. **b**, Illustration of the edge state acoustic wavefunction (hot color, below the green dashed line) and its 1D representation $\int dy\,|P_{\text{edge}}(x,y)|$^2 (above the green dashed line). Due to the absence of mirror symmetry on the edge, a finite geometry-induced edge polarization, p_{geom}, emerges.

Supplementary Note 5: Validity of the 2D approximation

In the main text, we have stated that to approximate the proposed 2D system, a quasi-2D experimental set-up is utilized, where the fabricated samples are cladded by acoustically rigid boards from the above and below. The vertical height of the samples is set as 1 cm, such that at the measurement frequencies, only zero-th order mode is excited. This ensures the 2D approximation is applicable. In Supplementary Figure 6a, we compare the bulk band structures of the 2D SC and that of the quasi-2D SC with finite height of 1 cm. Good agreements between the two band structures indicate that the quasi-2D model indeed well captures the quantitative features of the proposed system. In addition, we present the acoustic field profiles of the eigen-states at the M point, in Supplementary Figure 6b and S6c, respectively, for the 2D and the quasi-2D models. These field profiles again confirm that only zero-th order modes are excited in the quasi-2D model and it essentially carries the same physics as the 2D model.

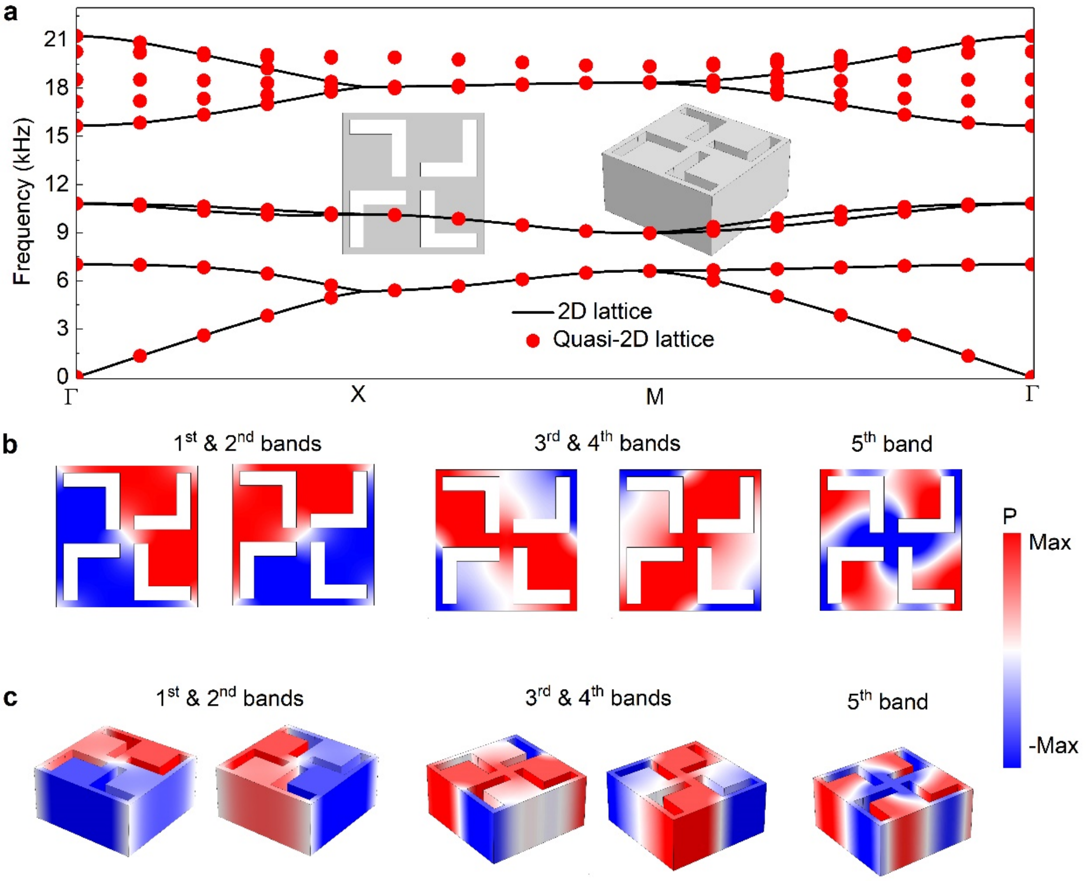

Supplementary Figure 6 | Validity of the 2D approximation. a, Bulk band structures of the 2D and the quasi-2D models (insets represent the unit cells of the models), with the acoustic field profiles of the eigen-modes at the M point shown in **b,** for the 2D model and in **c,** for the quasi-2D model. They exhibit good consistence with each other.

Supplementary Note 6: Topological transition for the quadrupole moment

In the main text, we have demonstrated that a topological transition for the quadrupole moment occurs when the mirror symmetries are introduced into the system. To illustrate the relation between symmetry and topology in our acoustic metacrystals, we study the topological phase transition for the second acoustic gap which is solely triggered by tuning the geometry of the metacrystal. As shown in the main text, the continuous geometry transition is as follows: first decrease the arch height h of the arch-shaped scatterers, then reduce the arm-length l of the scatterers, and finally increase the arm-width w of the scatterers. Along this transformation, at $h = 0$, the symmetry of the SC undergoes a transition from the nonsymmorphic $p4g$ wallpaper symmetry to symmorphic C_{4v} point group symmetry. Supplementary Figure 7f gives the topological phase diagram of the acoustic metacrystal. Through the geometry transition

from the $p4g$ wallpaper group to the C_{4v} point group, the second band gap undergoes a transition from a quadrupole topological to trivial. The topological transition takes place exactly at the geometry transition point which reflect elegantly the interplay between symmetry and topology. As a consequence, the corner states merge into the bulk bands at the topological transition, while they disappear when a trivial band gap is open for the C_{4v} sonic crystals, as shown in Supplementary Figure 7f.

The topological transition is directly reflected by the gapless Wannier bands. In Supplementary Figure 7, we present the Wannier bands for a topological SC (different from the one presented in Fig. 1 of the main text) and a trivial SC. Notice that as the arch-height h reduces, the Wannier bands in the difference sectors move towards ± 0.5. When $h \rightarrow 0$, both the acoustic band gap (between the fourth and the fifth bands) and the Wannier band gap closes, signifying a topological transition. By further reducing the arm length l and then increasing the arm width w, a trivial band gap is opened where the Wannier bands remain gapless (Supplementary Figure 7e). The gapless nature of the Wannier band is connected to the fact that for $h \rightarrow 0$, the mirror symmetries, $M_x := (x, y) \rightarrow (-x, y)$ and $M_y := (x, y) \rightarrow (x, -y)$, emerge. As shown in Supplementary references [1, 2], commutative mirror symmetries (i.e., $M_x M_y = M_y M_x$) lead to gapless Wannier bands. These results show that the non-commutativity of the glide symmetries (i.e., $G_x G_y \neq G_y G_x$) is essential for the emergence of the gapped Wannier bands and the quadrupole topology.

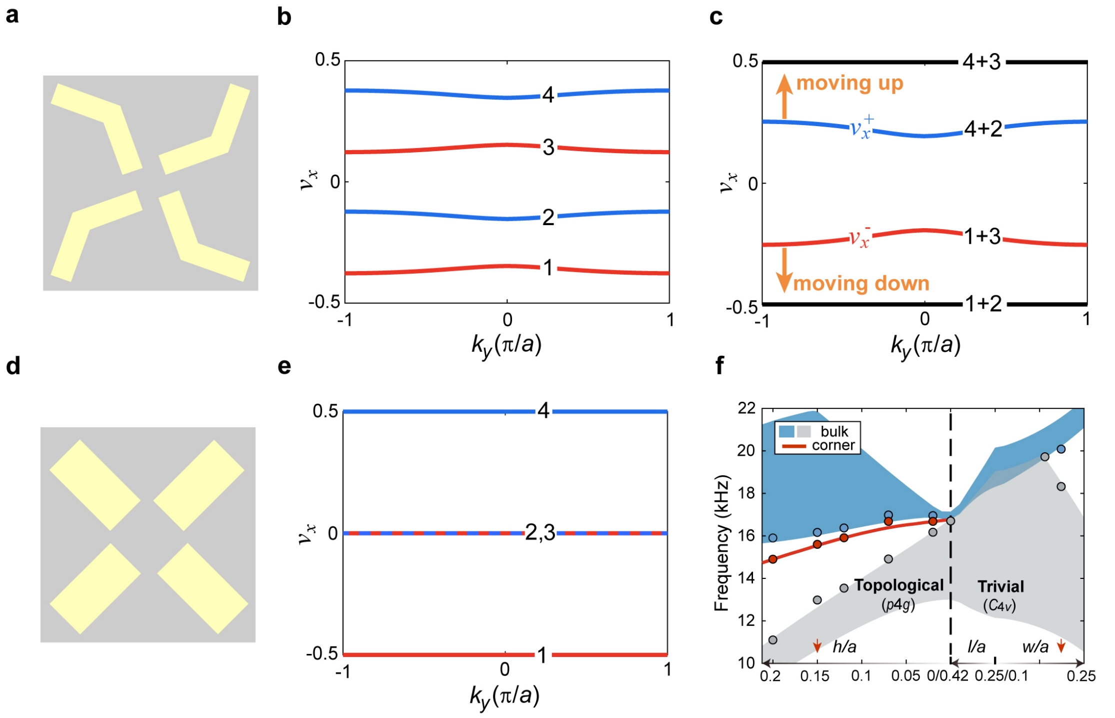

Supplementary Figure 7 | Wannier bands for the topological (trivial) SC and topological phase transition in the second band gap. a, b and c, Respectively, the unit-cell structure, Wannier bands and the Wannier bands in different sectors for the SC with $h = 0.15a$, $l = 0.42a$ and $w = 0.1a$.

The orange arrows with 'moving up' and 'moving down' signify that '4-3' and '1-2' sectors move to 0.5 and -0.5, respectively, for $h \to 0$. **d and e,** Respectively, the unit-cell structure and Wannier bands for the sonic crystal with $h = 0$, $l = 0.25a$ and $w = 0.21a$. **f,** Topological phase transition induced by continuously tuning the geometry, i.e., first reduce the arch height h, then reduce the length l, and finally increase the width w. The band diagram shows the bulk band gap closing and reopening, while the corner states disappear in the trivial region. The corner states are calculated using a box-shaped finite structure, as illustrated in the inset. The topological transition takes place when the arch height vanishes at $h = 0$. The points represent experimental data for a sample with 10×10 unit-cells. The bulk band regions are calculated using infinite (periodic) structures, while the corner states are calculated using a finite structure with 10×10 unit-cells.

To study the evolution of the corner states during the topological transition, we conduct the eigen-evaluations on a box-shaped finite structure with 10×10 unit-cells, surrounded by an air layer with thickness of $0.25a$ and enclosed by hard-wall boundaries. A phase diagram depicting the frequencies of the corner, edge and bulk states is provided in Supplementary Figure 7f. It is shown that when $h \to 0$ (with $l = 0.42a$ and $w = 0.1a$), the corner states merge into the bulk bands due to the topological transition. This is a hallmark feature of quadrupole topology that the corner states are induced directly by the bulk quadrupole moment and disappear when the bulk quadrupole moment vanishes. The width of the air gap is set to ensure that the topological corner states merge into the bulk bands precisely at the transition point. In this way, other non-topological mechanisms that may induce corner states can be excluded. On the other hand, in the topologically trivial regime where mirror symmetries emerge and the bulk band gap is reopened, there is no corner states in the band gap.

Experimentally, we fabricate several SCs with different geometries and measure the bulk band gap as well as the frequency of the corner states (if they exist). The detailed experimental measurements are illustrated in Supplementary Figure 8 and discussed in the paragraph below. The geometric transformation of the SCs is illustrated in Fig. 2a in the main text, with a transition from the wallpaper $p4g$ symmetry to the C_{4v} point group symmetry. The measured bulk band gaps (blue and gray points in Supplementary Figure 7f for upper and lower band-edges, respectively) for those SCs agree fairly well with the calculation. Both experiments and calculation show that the corner states merge into the bulk bands at exactly the topological transition point where the symmetry transition from $p4g$ group to C_{4v} group takes place. We also confirm experimentally that after entering into the gapless regime and reopening of the bulk band gap with trivial topology, the corner states do not appear again. This phase diagram confirms the quadrupole topology by establishing the connection between the bulk quadrupole topology and the emergent corner states from both experimental and

theoretical aspects.

The experimental measurements on the phase diagram are performed by measuring the frequency responses of different types of boundary states (i.e., the corner and edge states) and bulk states under the excitation of point-like sources. Specifically, we conduct three types of pump-probe measurements for each geometric combination (there are eight combinations in total), whose results are shown in Supplementary Figure 8. The source and detection locations for each measurement are illustrated in the insets. From each measurement, we extract the peak frequencies for the excited corner, edge and bulk states (indicated by the colored arrows in different subfigures for different geometries) and provide these frequencies in the phase diagram in Fig. 2a of the main text using colored dots. It is seen that the experimental measurements are consistent with the simulated results, with a small discrepancy resulted from the fabrication and measurement errors.

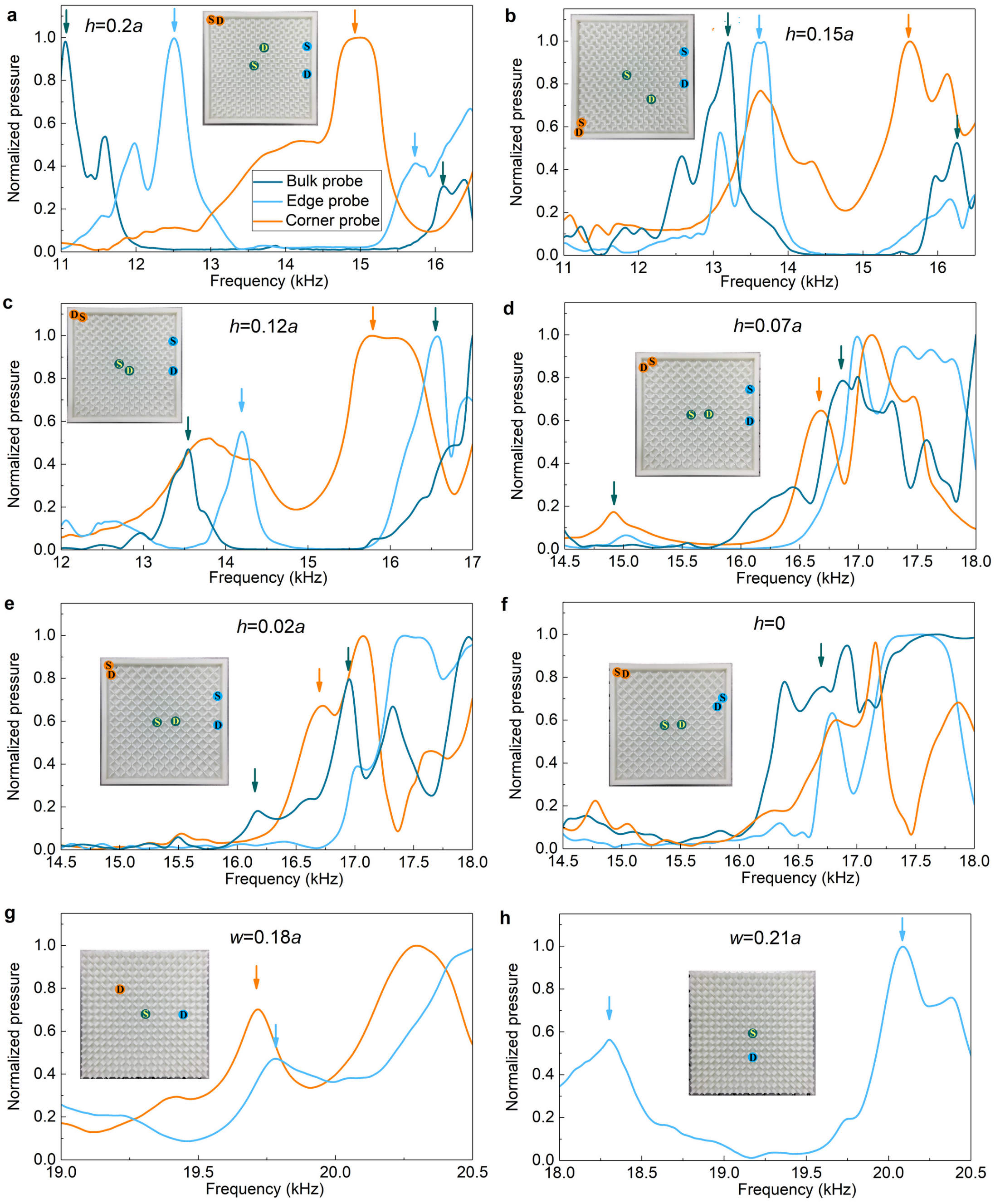

Supplementary Figure 8 | Experimental transmission spectra measured for eight geometry combinations on the phase diagram. a, The unit-cell geometry parameters are $h = 0.2a$, $l = 0.42a$ and $w = 0.1a$. **b**, The unit-cell geometry parameters are $h = 0.15a$, $l = 0.42a$ and $w = 0.1a$. **c**, The unit-cell geometry parameters are $h = 0.12a$, $l = 0.42a$ and $w = 0.1a$. **d**, The unit-cell geometry parameters are $h = 0.07a$, $l = 0.42a$ and $w = 0.1a$. **e**, The unit-cell geometry parameters are $h = 0.02a$, $l = 0.42a$ and $w = 0.1a$. **f**, The unit-cell geometry parameters are $h = 0$, $l = 0.42a$ and $w = 0.1a$. **g**, The unit-cell geometry parameters are $h = 0$, $l = 0.25a$ and $w = 0.18a$. **h**, The unit-cell geometry parameters are $h = 0$, $l = 0.25a$ and $w = 0.21a$. For all these figures, $a = 2$ cm. The source and detection locations for each measurement are illustrated in the insets. The peak frequencies for the excited corner, edge and bulk states are indicated by the colored arrows, corresponding to the colored dots in Supplementary Figure 7f.

In addition, the normalized density of states (DOS) for different types of boundary and bulk states is presented in Fig. 2 of the main text to provide explicit evidence on how the topological corner states merge into the bulk states accompanying the topological transition. Here, we give a brief description on the procedure to calculate the DOS. Using the software COMSOL Multiphysics, we conduct eigen-evaluations on the box-shaped finite structures (the same as that used in Fig. 2a of the main text). We make sure enough eigen-states are obtained such that their eigen-frequencies cover the frequency range of interest. The nature of the ith eigen-state with eigen-frequency f_i (i.e., whether it is a corner, edge or bulk state) is determined by the spatial distribution of its wavefunction. It is characterized by the energy proportion in each representative domain j (illustrated in the inset in Fig. 2c of the main text for the corner, edge and bulk evaluations) in the box-shaped supercell. We use A_i^j to denote the energy proportion, which is defined as $A_i^j = \frac{I_i^j}{I_i^{\mathrm{All}}}$, where I_i^j indicates the integration of $|P|^2$ over the jth representative corner/edge/bulk domain and I_i^{All} indicates the integration over the whole domain of the supercell. Then the DOS can be accordingly calculated by $\mathrm{DOS}^j(\omega) = \sum_i \frac{1}{\pi} \frac{\Gamma \cdot A_i^j}{(\omega - f_i)^2 + \Gamma^2}$, where ω represents the central point of the frequency interval where the calculation is performed and Γ is the length of the frequency interval. We need to point out that this frequency interval has to be small enough in order to fully resolve the obtained eigen-states. A Lorentzian fitting is applied by taking dissipation into consideration. As shown in Fig. 2 of the main text, the corner, edge and bulk states can be conveniently distinguished by their representative DOS peaks, which also provide explicit evidence to the topological transition when the corner peak starts to mingle with the edge and bulk peaks. We further present in Supplementary Figure 9 the acoustic pressure fields of the corner states with $h = 0.010a$, $h = 0.005a$, and $h = 0$. It is seen that the acoustic fields are indeed going from the localized corner state to the extended bulk state during the topological transition process. The direct transition from the corner states to the bulk states is a manifestation of the bulk-corner correspondence, namely that the emergence and disappearance of the corner states are solely due to the bulk quadrupole topology.

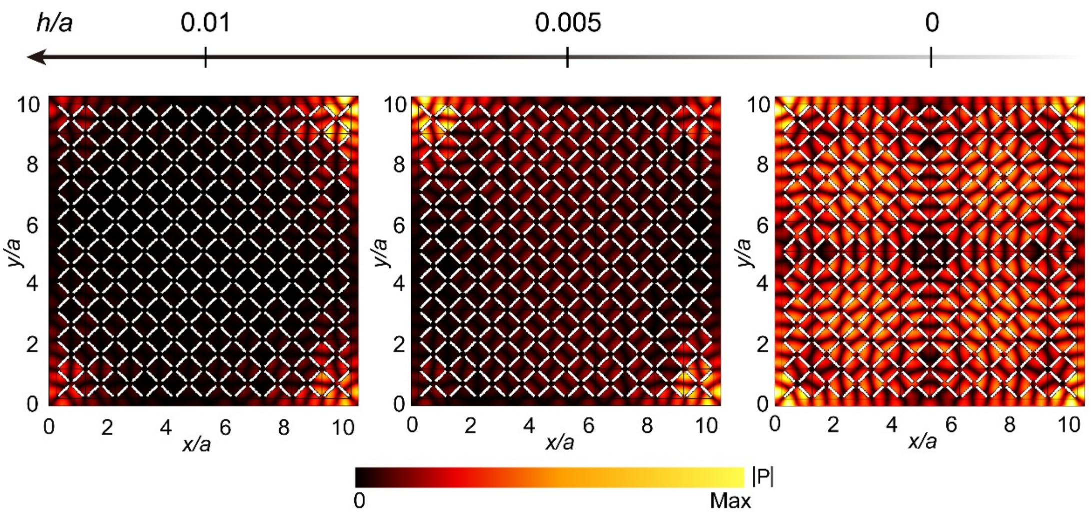

Supplementary Figure 9 | Acoustic pressure fields of the corner states as $h \to 0$. From left to right panels: $h = 0.010a$, $h = 0.005a$, and $h = 0$.

Supplementary Note 7: Robustness of the quadrupole topological edge and corner states

In this section, we perform numerical studies on the robustness of the quadrupole topological edge and corner states against two types of disorders: (i) defects that preserve the crystalline symmetry at the unit-cell level; (ii) defects that break the crystalline symmetry at the unit-cell level. The defect-free structure for the edge state simulations has a ribbon-like geometry with the topological SC of $5{\times}20$ unit cells enclosed by the hard-wall boundaries along the x-direction (as illustrated in Supplementary Figure 10a). The hard-walls and the SC are separated by two air layers of thickness $0.25a$. Along the y-direction, the boundaries are set as absorbers to avoid reflection at the boundaries. Here, we simulate the excitation of the edge state with a source below the detect (see Supplementary Figure 10a, left panel). For each simulated structure, Supplementary Figure 10 presents the schematic of the geometry (left panel), the acoustic pressure profile for one excitation frequency (middle panel), and the transmission spectra for both the edge-probe and the bulk-probe (right panel). A sample without defect is first studied as the benchmark. As shown in Supplementary Figure 10a, clear spectral features of edge states within the bulk band gap is observed. The band gap of the edge states is also faithfully reflected in the transmission spectrum. We then investigate how the defects affect the edge state propagation. Two types of defects are considered, including both symmetry-preserving defects (i.e., amplification of the structure in some unit-cells) and symmetry-breaking defects (i.e., rotation of the structure in some unit-cells). Specifically, Supplementary Figure 10b presents the effect of symmetry-preserving defects on the edge state propagation where the structures of

some unit-cells on the edge are enlarged by 10%. It is seen that the transmission spectra for both the edge and the bulk remain mostly unchanged. Supplementary Figure 10c presents the results when the defects are introduced slightly away from the edge, the effect of defects on the transmission spectra becomes even weaker. These simulation results confirm the robustness of the topological edge states against symmetry-preserving defects.

Supplementary Figures S10d-S10f show the effects of symmetry-breaking defects on the edge state propagation. Specifically, Supplementary Figure 10d presents the results when the unit-cell structure is rotated by 5° at three locations on the edge. Supplementary Figure 10e shows the results when the unit-cell structure is rotated by 5° at three locations slightly away from the edge. Supplementary Figure 10f indicates the results when both 5° and -10° unit-cell-rotations are introduced at six locations. From the results in all these figures, we find that the main spectral features such as the edge band gap are almost unchanged, while the detailed spectral features away from the edge gap may be modified. The robustness of the edge band gap is crucial to the bulk-edge-corner correspondence for the quadrupole topology. We thus conclude that the topological edge states are found in general to be robust against both symmetry-protecting and symmetry-breaking defects, with the effect of latter more significant than the former, as expected. We notice that even when the acoustic field distributions of the edge states are severely distorted by the defects, the spectral signature of the edge band gap is still sharp (see Supplementary Figure 10f).

We now study the effect of disorder on the corner states. In the corner state simulation, the defect-free structure is a box-shaped supercell with the topological SC of 10×10 unit cells, surrounded by the air layer of thickness $0.25a$ and enclosed by the hard-wall boundaries. We consider both the symmetry-preserving and symmetry-breaking defects. The results are presented in Supplementary Figure 11. The robustness of the corner state is characterized by its frequency stability in the transmission spectra. The set-up for the pump-probe transmission in the simulation is illustrated in Supplementary Figure 11a. From the figure, one can see that the frequency of the corner state is more robust against the symmetry-preserving defects introduced by amplifying the unit-cell structures (see Supplementary Figure 11b-d) than the symmetry-breaking defects introduced by rotating the unit-cell structures (see Supplementary Figure 11e-h). In the case of strong deformation (Supplementary Figure 11d), the defect can introduce a new defect mode (indicated by the blue arrow in the figure) in addition to the corner state, whose frequency, however, still changes negligibly. These results convincingly demonstrate the robustness of the corner state against symmetry-preserving disorders.

The symmetry-breaking perturbations, on the other hand, has strong effect on the frequency of the corner state. The frequency of the corner state can change up to 11%,

as shown in Supplementary Figure 11h. Nevertheless, the corner state maintains its main features, i.e., localized at the corner and decayed rapidly into the bulk and edges, regardless of the existence of multiple defects.

In addition to the simulations, we also perform experimental study on the robustness of the corner state against an amplification of the corner cell by 10% (Supplementary Figure 12b) and a rotation of the corner cell by 10° (Supplementary Figure 12c). The case without disorders is also presented for comparison (see Supplementary Figure 12a). The experimental results are consistent with the simulations: the corner state does not disappear under these distortions and is more robust against the symmetry-protecting defect (amplification of the unit-cell structure) than the symmetry-breaking defect (rotation of the unit-cell structure).

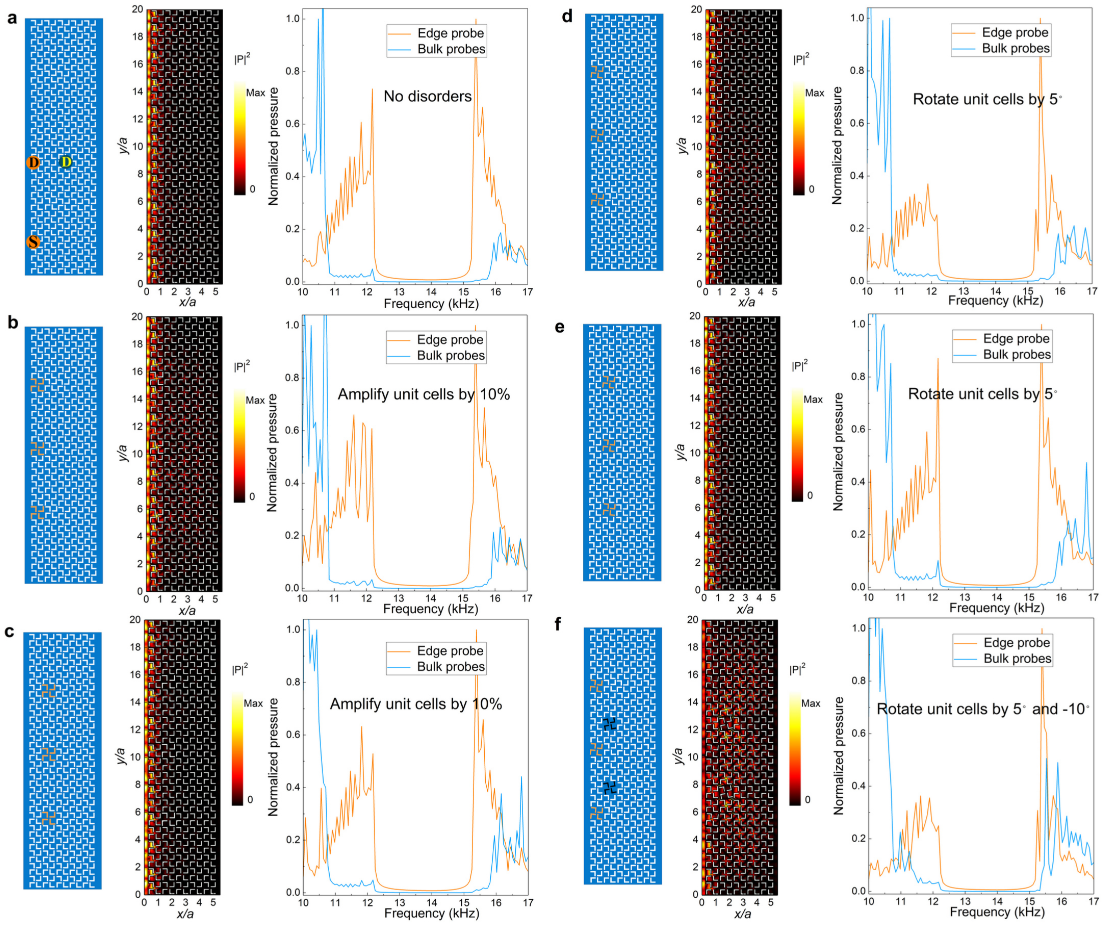

Supplementary Figure 10 | Simulation on the robustness of the quadrupole edge states. A defect-free structure is studied as benchmark in **a,** and two types of defects are considered, including glide-symmetry-protecting defects (i.e., amplification of unit cells in **b** and **c**) and glide-symmetry-breaking defects (i.e., rotation of unit cells in **d-f**). These deformations are sketched in color in the left panels. The middle panels depict the acoustic pressure profile at one excitation frequency and the right panels present the transmission spectra for both the edge (orange curves) and bulk (blue curves) probes. The S and D in the left of **a** indicate the source and detection in the transmission

simulation.

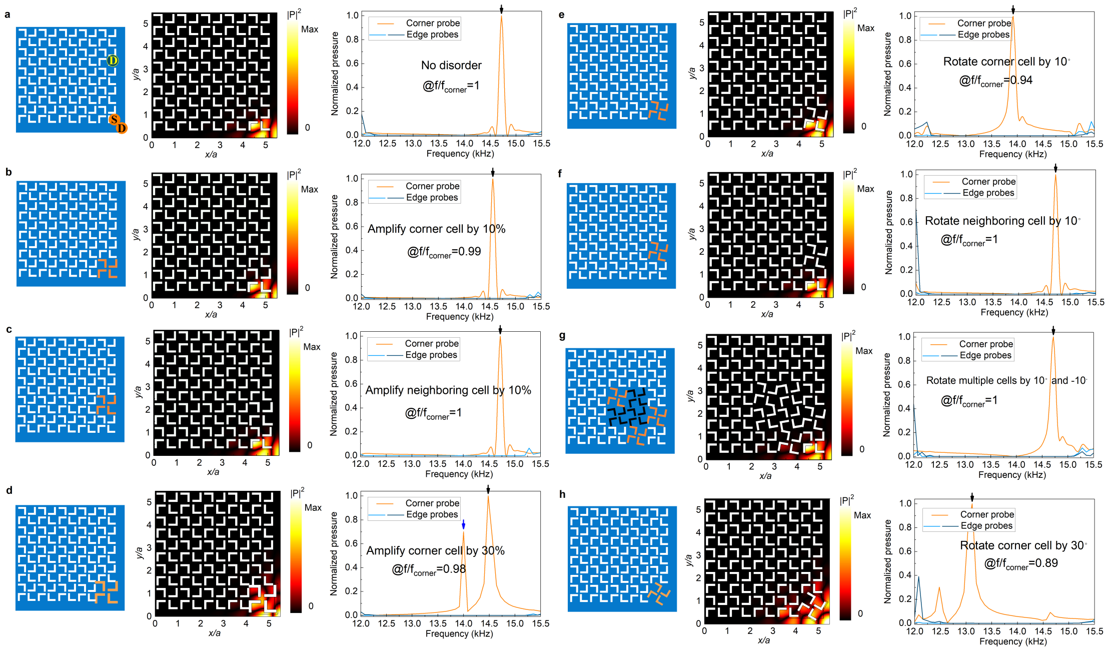

Supplementary Figure 11 | Simulation on the robustness of the quadrupole corner states. The robustness of the corner state is characterized by its frequency stability under perturbations, which is marked in each transmission spectrum plot (in both black arrows and the ratio of the shifted frequency to the original one), along with the notation for the type of defects (with specific deformations). Two types of defects are studied: amplification of the structure in some unit-cells (symmetry-preserving) and rotation of the structure in some unit-cells (symmetry-breaking). **a,** The original structure. **b-d**, Symmetry-preserving disorders. **e-h**, Symmetry-breaking disorders.

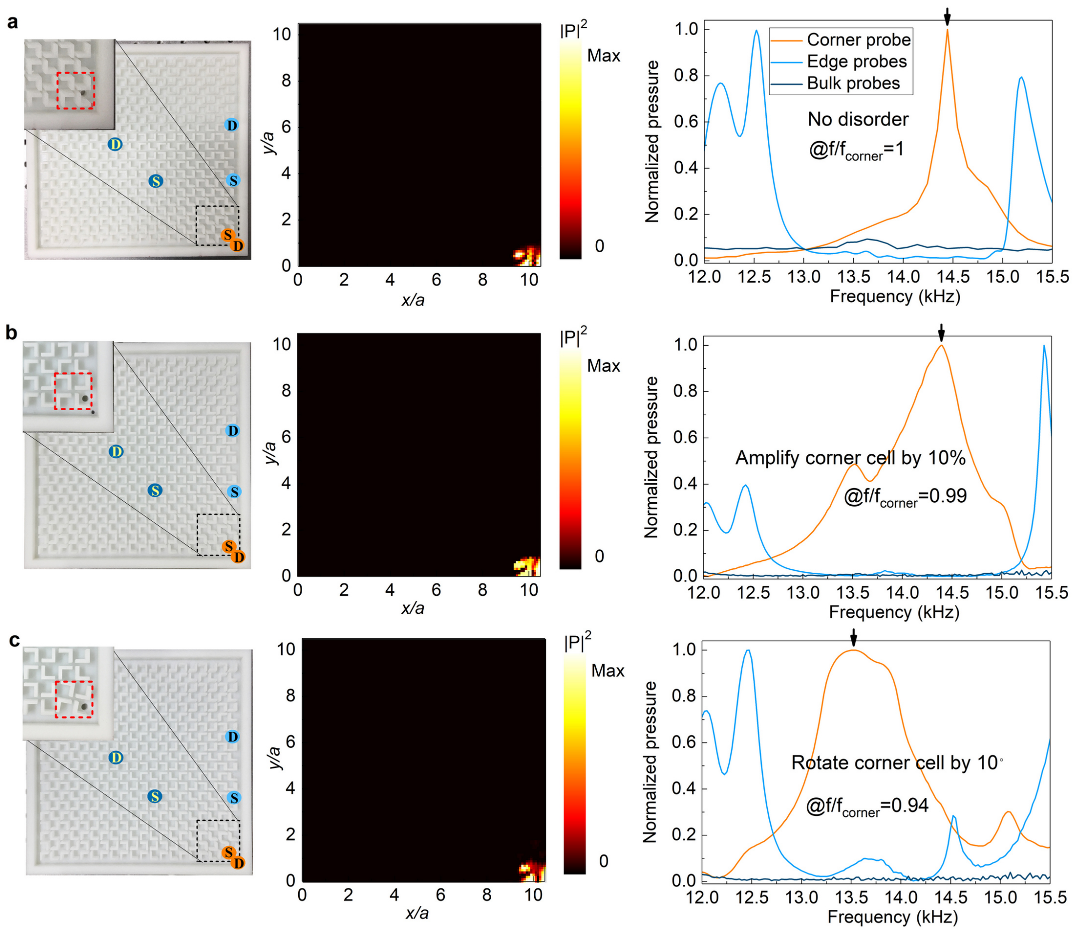

Supplementary Figure 12 | Experimental study on the robustness of the quadrupole corner states. The clean structure is studied as comparison in **a.** The robustness of the corner state against an amplification of the corner cell in **b** and a rotation of the corner cell in **c** is presented. The left panels depict the fabricated samples used in the experiments, the middle panels show the measured corner state field distributions and the right panels present the measured transmissions spectra for the corner, edge and bulk probes. These results are consistent with the simulation presented in Supplementary Figure 11.

Supplementary Note 8: Measurements of the quadrupole edge gap and the transmission spectra for various pump-probe configurations

The experimental data of the edge dispersions are obtained by measuring the acoustic pressure fields, P, along the edge, which are further Fourier-transformed into the momentum domain. The obtained data F_P=$|\text{fft}(P)|^2$ are discretized functions of frequency ω and wave vector k_y (assume the edge is along the y direction) and are normalized to give $\sum_{k_y} F_P(\omega, k_y) = 1$. The color maps (hot color) in the Figs. 3 and 5 in the main text are plotted using $F_P(\omega, k_y)$.

The transmission measurements of the acoustic fields as function of source frequency around the edge gap are further presented in Supplementary Figure 13 for the edge states in the quadrupole topological gap. In the measurement, two points on the edge, located at the opposite sides of the source position (at the center of the edge, as shown in the inset of Supplementary Figure 13), are probed. The measurements are conducted using the way detailed in the method section. As shown in Supplementary Figure 13, both probes find two pressure peaks around the frequencies of 12.3 kHz and 15.2 kHz, which clearly mark the band edges, consistent with the measurements in Fig. 3a. In between these frequencies, there exists the edge gap (shaded area).

The response spectra of the bulk-probe, edge-probe and corner-probe are presented in Supplementary Figure 14 without normalization. These unnormalized data indicate that the peak corner response is more than 80 times larger than the bulk-response, which manifests the strong enhancement of the acoustic wave intensity at the corner due to the existence of the corner states. Similar study is also performed in simulation. The results are shown in Supplementary Figure 15, which exhibit similar spectral features with both Fig. 4b in the main text and Supplementary Figure 14, confirming the existence of the corner, edge and bulk states in a single acoustic crystal. Specifically, the simulation results in Supplementary Figure 15 give a bulk spectral gap comparable with the experimental results. The emergence of the edge transmission peaks in the bulk band gap and the corner transmission peak within the band gap of the edge states is clearly visible. The frequencies of the corner states and the edge bands are comparable with the the experimental transmission spectra as well. Some detailed features are different. For instance, the corner resonance in the simulation is at 14.7 kHz, while in the experiments it is 14.5 kHz. In addition, the edge spectral gap in the simulation is slightly larger than the measured edge spectral gap in experiments. Most notably, the resonances for both the edge and corner states are much broader in the experiments than in the simulation. These differences are mainly because of the following reasons: (1) In the simulation, we use point sources and point detectors for the excitation and detection of acoustic waves. However, in experiments, the sources and the detectors have finite size (their genuine geometric features are hard to model in the simulation). (2) Acoustic waves have dissipation during the propagation processes in realistic ambient environment, which we did not include in the simulation. (3) We do 2D simulations, while the experiments is done in a quasi-2D system. (4) The speed of sound and the mass density of air may be different in the laboratory environment compared with the ideal set-up in the simulation.

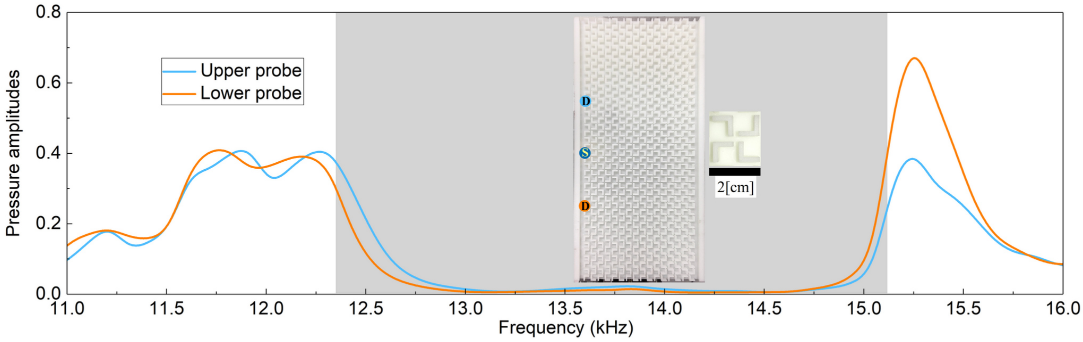

Supplementary Figure 13 | Measurements of the edge gap. The transmission spectra of the acoustic fields probed on the edge around the edge gaps are presented. Two locations at the opposite sides of the source are probed, indicated by the blue and orange curves separately. The edge gap region is shaded. The inset indicates the sample and the set-up utilized to conduct the measurements.

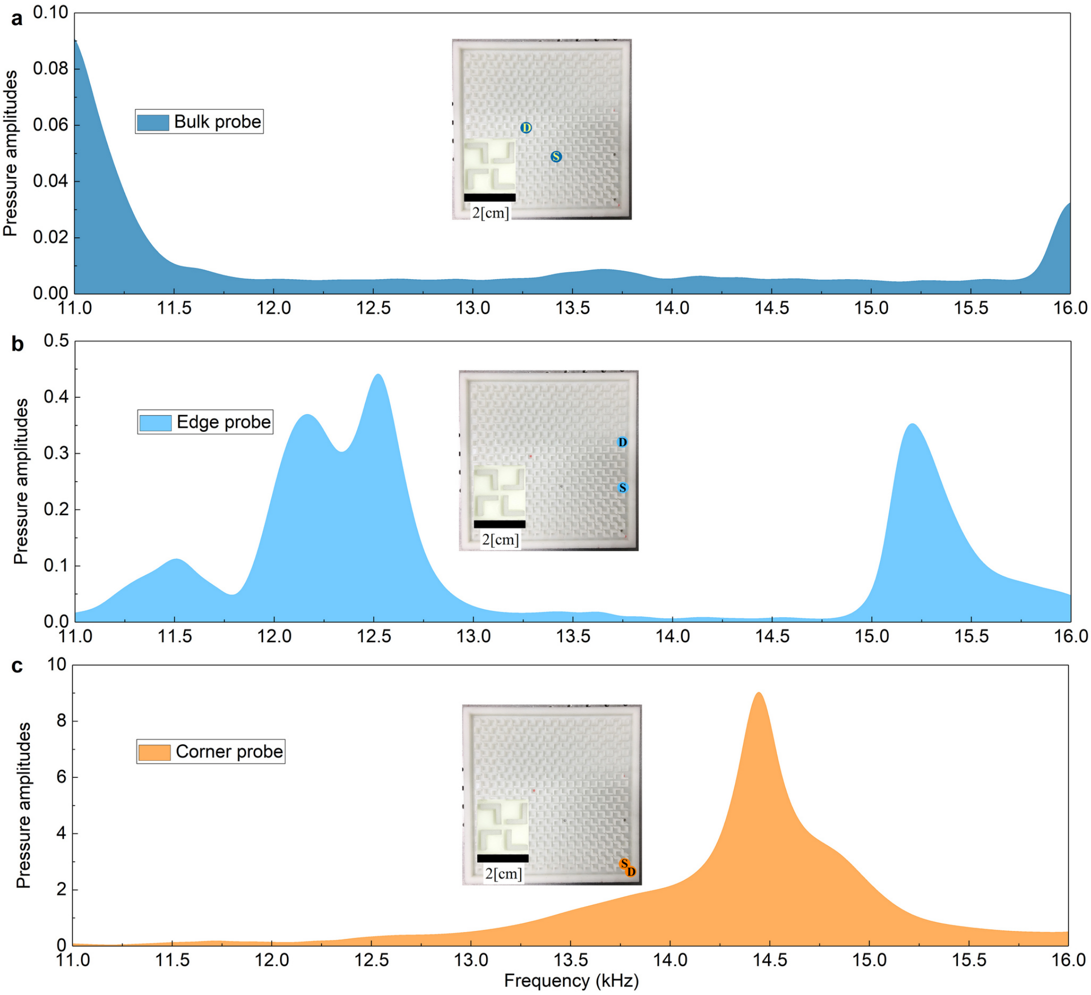

Supplementary Figure 14 | Transmission spectra of the bulk, edge and corner measurements without normalization. a-c, The same transmission measurements as that in Fig. 4b in the main text, but with the un-normalized, original transmission spectra.

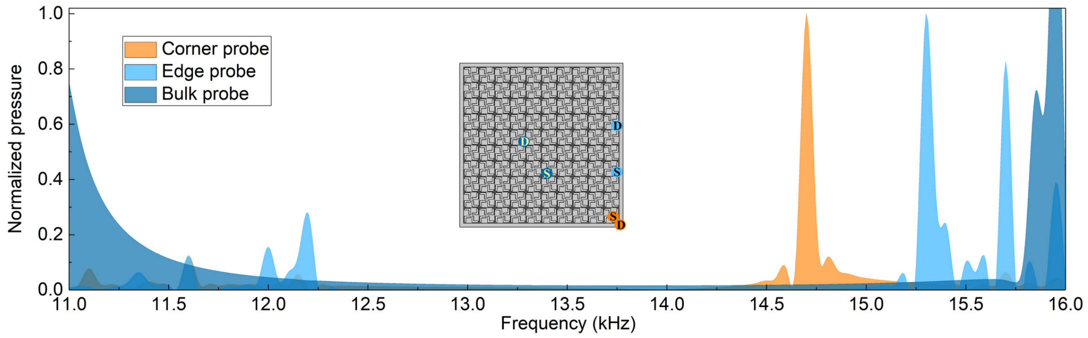

Supplementary Figure 15 | Transmission spectra of the bulk, edge and corner probes with normalization from simulation. The same set-up in Fig. 4b in the main text is used in the simulation. Point source and point detections are used in the simulation to approximately model the finite-sized source and detections used in the experiments. The main spectral features agree well with Fig. 4b in the main text, while the discrepancies are due to the approximation of the point source and detections, as well as other differences between the simulations and experiments (see Methods for details).

Supplementary Note 9: Real-part of the acoustic field for the corner state from experiments

Throughout this work, we have reported the calculation and measurements of the absolute values for the acoustic pressure fields for the corner states. Here, we show the real-part of the pressure fields in Supplementary Figure 16 for the corner state in Fig. 4c of the main text, compared with its simulated counterpart. We emphasize that since each corner supports only one corner state and the four corner states are connected by the C_4 rotation symmetry, only one corner is investigated here. The measured real-part in our experiments agrees well with the simulation results.

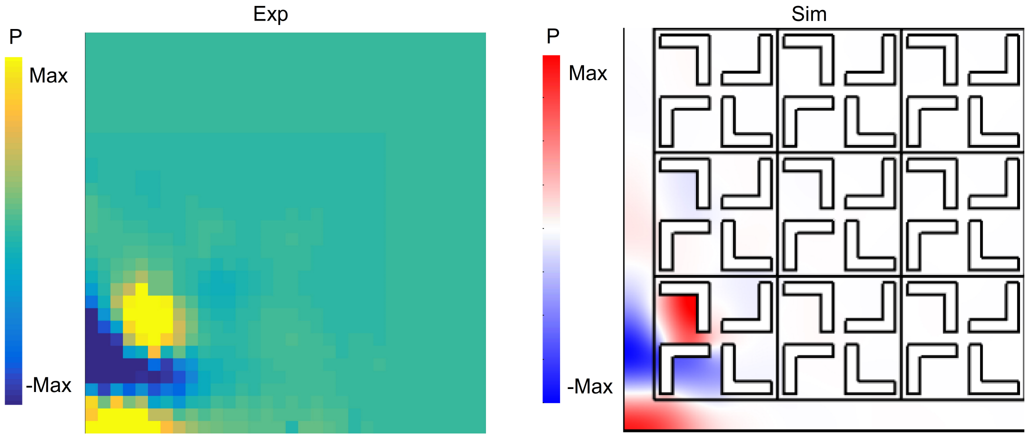

Supplementary Figure 16 | Real-part of the acoustic pressure profiles for the corner state reported in Fig. 4c of the main text. A simulated result is also provided for comparison.

Supplementary Note 10: Calculated edge and corner states and topological transition in the second band gap with different widths of the air-channel

In the measurements and calculations of the edge and corner states in the second band gap, we set a hard-wall boundary enclosing the SC with an air channel separating them to construct physical edges and corners for the acoustic waves. The width of the air-channel is determined as $0.25a$, such that the waveguide effect of the air channel can be eliminated from the observation and the corner states are guaranteed to merge into the bulk bands precisely at the topological transition point. In this way, other trivial mechanisms that may induce corner states can be excluded. Nevertheless, the topological edge and corner states exist for other widths of the air-channel as well. To provide the evidence, here we calculate the band structures for the ribbon-like supercells and the eigen-spectra for the box-shaped supercells, where the same unit-cell geometry parameters in the main text are used while the width of the air-channel varies from $0.2a$ to $0.5a$, which is still below the cut-off limit for the waveguide effects to appear in our frequency regime of interest. The results are shown in Supplementary Figure 17, with the upper panels for the band structures and the lower panels for the eigen-spectra. It is seen that the topological edge and corner states remain existent in the same way as that reported in the main text.

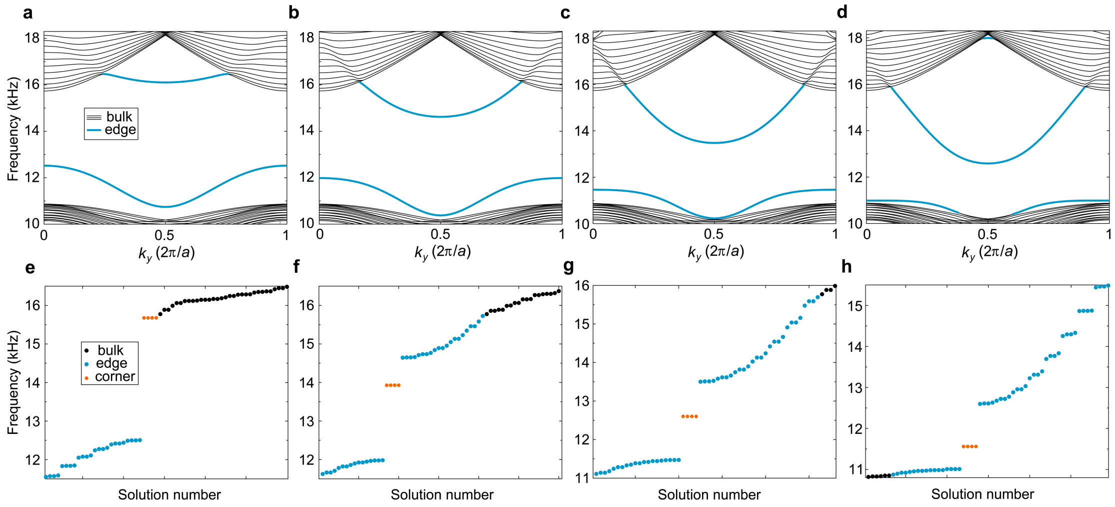

Supplementary Figure 17 | Topological edge and corner states in the second band gap with different widths of the air-channel. Simulated band structures of the ribbon-like supercells with the air-channel of width **a,** $0.2a$, **b,** $0.3a$, **c,** $0.4a$ and **d,** $0.5a$. **e-h,** The corresponding eigen-spectra for the box-shaped supercells. Unit-cell geometry parameters are $h = 0.21a$, $l = 0.42a$, $w = 0.1a$ and $a = 2$cm.

Then, taking the width $0.4a$ as an example, we calculate the local DOS for corner, edge, and bulk regions as that in Fig. 2 for certain geometry parameters, demonstrating

the topological transition in the second band gap. As shown in Supplementary Figure 18, the sharp peak of the corner DOS become indistinguishable when $h = 0$, indicating the corner states disappear when the quadrupole moment vanishes and the bulk band gap closes. This is consistent with the conclusion in the main text.

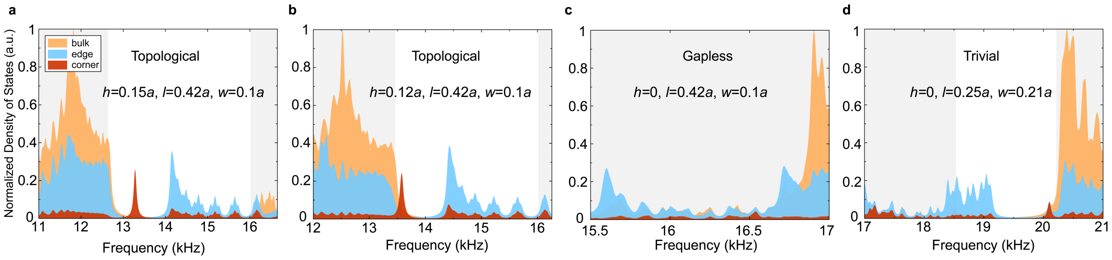

Supplementary Figure 18 | Calculated local density-of-states around the second band gap with the air-channel of width $0.4a$. **a**, Geometry parameters of the unit-cell: $h = 0.15a$, $l = 0.42a$, $w = 0.1a$. **b**, Geometry parameters of the unit-cell: $h = 0.12a$, $l = 0.42a$, $w = 0.1a$. **c**, Geometry parameters of the unit-cell: $h = 0$, $l = 0.42a$, $w = 0.1a$. **d**, Geometry parameters of the unit-cell: $h = 0$, $l = 0.25a$, $w = 0.21a$. For all figures, $a = 2$cm.

Supplementary Note 11: Topological transition for the first bulk band gap with different geometry parameters

In the main text, the unit-cell geometry parameters are chosen as $h = 0.21a$, $l = 0.42a$, $w = 0.1a$ and $a = 2$cm for the sake of experimental fabrications. However, as shown in Figs. 5a and 5b of the main text, under these parameters, non-smooth frequency change occurs in the phase diagram for the first bulk band gap at the M point during the rotation of the scatterers, and the acoustic bulk bands become dispersionless along the ΓXline when the rotation angle θ equals to $90°$. This is because the arch-shaped scatterers in the neighboring cells touch with each other, forming connected hard walls along the y-direction and forbidding the acoustic wave from propagating along the x-direction. Here, to show that this flat-band feature disappears for smaller scatterers and it does not affect the topological transition in the first band gap, we consider a smaller scatterer with geometry of $h = 0.18a$, $l = 0.35a$ and $w = 0.1a$, which guarantee the scatterers are well separated from each other for any rotation angle. In Supplementary Figure 19, we present the same results as that in Figs. 5a and 5b in the main text, only for the smaller scatterers. It is observed that the evolution curves indeed become smooth and the acoustic bands are not flat any more. More importantly, the topological transition depicted in Supplementary Figure 19 is exactly the same as that reported in Fig. 5, suggesting that the geometric difference does not affect the topological properties of the first band gap.

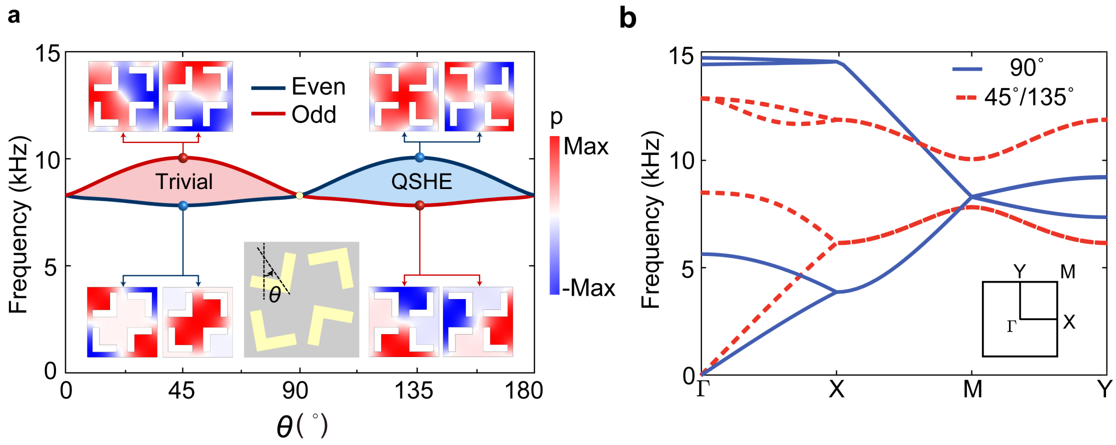

Supplementary Figure 19 | Same studies as that in Figs. 5a and 5b in the main text, only for the unit-cell geometry parameters of $h = 0.18a$, $l = 0.35a$ and $w = 0.1a$. a, The topological phase diagram for the first acoustic band gap. **b**, Acoustic band structures for three of the rotation angles.

Supplementary Note 12: Hamiltonian theory of the Dirac point and the first acoustic band gap

In Fig. 5a of the main text, we present the phase transition process of the SC in the first band gap, which is signaled by an emergent four-fold degenerate Dirac point (DP) at the M point for $\theta = 90°$, as shown in Fig. 5b in the main text. The aim of this section is to use the $\mathbf{k} \cdot \mathbf{P}$ theory to understand the dispersions around the DP. The acoustic wave propagation in a 2D SC is described by the following time harmonic wave equation

$$\kappa(\mathbf{r})\nabla \cdot [\rho^{-1}(\mathbf{r})\nabla\psi(\mathbf{r})] + \omega^2\psi(\mathbf{r}) = 0, \tag{20}$$

where $\psi(\mathbf{r})$ represents the pressure field, with $\kappa(\mathbf{r})$ and $\rho(\mathbf{r})$ denoting the constitutive bulk modulus and mass density, respectively. Supplementary Equation (20) can be further simplified to an eigen-value problem by applying the periodic boundary condition of the 2D SC,

$$H\psi_{n,\mathbf{k}}(\mathbf{r}) = \omega_{n,\mathbf{k}}^2\kappa^{-1}(\mathbf{r})\psi_{n,\mathbf{k}}(\mathbf{r}), \tag{21}$$

where $H = -\nabla \cdot [\rho^{-1}(\mathbf{r})\nabla]$ is the Hamiltonian. Here $\psi_{n,\mathbf{k}}(\mathbf{r})$ and $\omega_{n,\mathbf{k}}$, respectively, represent the wavefunction (for the pressure field) and the eigen-frequency of the nth Bloch band with wavevector $\mathbf{k}$. The eigen-states are orthogonal, following the normalization rules $\delta_{nn'} = \int_{u.c.} \psi_{n,\mathbf{k}}^*(\mathbf{r})\, \kappa^{-1}(\mathbf{r})\psi_{n',\mathbf{k}}(\mathbf{r})d\mathbf{r}$, where the integral runs over the unit cell ($u.c.$).

Applying the $\mathbf{k}\cdot\mathbf{P}$ theory, $\psi_{n,\mathbf{k}}(\mathbf{r})$ can be expanded by the Bloch wavefunctions at the M point through $\psi_{n,\mathbf{k}}(\mathbf{r}) = e^{i\mathbf{q}\cdot\mathbf{r}}\sum_{n'} C_{n,n'}\psi_{n',\mathbf{K}}(\mathbf{r})$, where $\boldsymbol{q} = \mathbf{k} - \mathbf{K}$ with $\mathbf{K} = (\frac{\pi}{a}, \frac{\pi}{a})$ and $C_{n,n'}$ are the expansion coefficients. The wavevector difference $\mathbf{q}$ is treated as a small quantity in the $\mathbf{k}\cdot\mathbf{P}$ theory. Direct calculation yields the following $\mathbf{k}\cdot\mathbf{P}$ Hamiltonian,

$$H_{nn'} = \omega_n^2\delta_{nn'} + \mathbf{q}\cdot\mathbf{P}_{nn'} + O(\mathbf{q}^2), \tag{22}$$

with ω_n the eigen-frequency of the nth band at the DP. Here we keep only up to the linear terms of $\mathbf{q}$, while the high-order terms are omitted. The matrix element of $\mathbf{P}_{nn'}$ is given by

$$\mathbf{P}_{nn'} = \int_{u.c.} \psi_{n,\mathbf{K}}^*(\mathbf{r})\{-i[2\rho^{-1}(\mathbf{r})\nabla + \nabla\rho^{-1}(\mathbf{r})]\}\psi_{n',\mathbf{K}}(\mathbf{r})d\mathbf{r}. \tag{23}$$

It should be noticed that $\mathbf{P}_{nn'}$ is nonzero only when n and n' bands are of different parities. In our system, around the DP, there are four states interacting with each other, where two of them are p-states with odd parity (see Fig. 5a in the main text), which we denote as $|p_x\rangle$ and $|p_y\rangle$, while the other two states are of even parity (see Fig. 5a in the main text), which are labeled as $|s\rangle$ and $|d\rangle$. Based on the symmetries of these four states, the $\mathbf{q}$-linear term in the basis of $(|s\rangle,|d\rangle,|p_x\rangle,|p_y\rangle)^{\mathrm{T}}$ is deduced as

$$q_xP_x + q_yP_y = \begin{pmatrix} 0 & 0 & aq_x & bq_y \\ 0 & 0 & cq_y & dq_x \\ a^*q_x & b^*q_y & 0 & 0 \\ c^*q_y & d^*q_x & 0 & 0 \end{pmatrix}. \tag{24}$$

Since the other acoustic bands are far away from the DP in frequency, the $\mathbf{k}\cdot\mathbf{P}$ theory can be restricted to the Hilbert space consisting of only the four states $|s\rangle,|d\rangle,|p_x\rangle,|p_y\rangle$, leading to the above $\mathbf{k}\cdot\mathbf{P}$ Hamiltonian.

The SC under consideration belongs to the wallpaper space group $p4g$, obeying the following glide operations,

$$G_x: (x, y) \to (\tfrac{1}{2} - x, \tfrac{1}{2} + y), \tag{25a}$$

and

$$G_y: (x, y) \to (\tfrac{1}{2} + x, \tfrac{1}{2} - y), \tag{25b}$$

where the lattice constant is assumed to be unity. We apply G_x and G_y to the four states $|s\rangle,|d\rangle,|p_x\rangle,|p_y\rangle$ and obtain $\langle p_y|G_x|p_x\rangle = -1$, $\langle p_x|G_x|p_y\rangle = 1$, $\langle d|G_x|s\rangle = -1$, $\langle s|G_x|d\rangle = 1$ for the G_x operation, as well as $\langle p_y|G_y|p_x\rangle = 1$, $\langle p_x|G_y|p_y\rangle = -1$, $\langle d|G_y|s\rangle = -1$, $\langle s|G_y|d\rangle = 1$ for the G_y operation (other matrix elements are zero). Here we consider anti-unitary operator Θ_x as an illustration, and the following discussed constraints on the Hamiltonian also apply to the symmetry operator Θ_y.

The invariance of the Hamiltonian under anti-unitary operator Θ_x implies

$$\Theta_x H(\mathbf{q}) \Theta_x^{-1} = H(\Theta_x \mathbf{q}). \tag{26}$$

Note that $\Theta_x \mathbf{q} = G_x T \mathbf{q} = -G_x \mathbf{q} = (q_x, -q_y)$, and T is time-reversal operator, which is represented as the complex conjugation (denoted by *K*) in the Hilbert space. Consequently, we find that

$$a = d^* \,; b = c^*. \tag{27}$$

According to the above, the effective Hamiltonian for a gapped Dirac point can be written in the basis of (|*s*>, |*d*>, |p_x>, |p_y>)^{T} as,

$$H(\mathbf{q}) = \begin{pmatrix} \omega_s^2 & 0 & a q_x & b q_y \\ 0 & \omega_d^2 & b^* q_y & a^* q_x \\ a^* q_x & b^* q_y & \omega_{p_x}^2 & 0 \\ b q_y & a q_x & 0 & \omega_{p_y}^2 \end{pmatrix}. \tag{28}$$

At the M point, the doubly degenerate states *s*> and |*d*>, as well as, |p_x> and |p_y> further hybridize into the pseudospin-up states, |d_+>= $\frac{1}{\sqrt{2}}$ (|*s*>+*i*|*d*>) and |p_+>= $\frac{1}{\sqrt{2}}$ (|p_x>+*i*|p_y>), and, the pseudospin-down states, |d_->= $\frac{1}{\sqrt{2}}$ (|*s*>-*i*|*d*>) and |p_->=$\frac{1}{\sqrt{2}}$(|p_x>-*i*|p_y>). Then, the Hamiltonian can be rewritten in the basis of (|d_+>, |p_+>, |d_->, |p_->)^{T} as,

$$H_{BHZ} = SHS^+ = \begin{pmatrix} M_+ & N \\ N^* & M_- \end{pmatrix}, \tag{29}$$

where $S = \frac{1}{\sqrt{2}} \begin{pmatrix} 1 & i & 0 & 0 \\ 0 & 0 & 1 & i \\ 1 & -i & 0 & 0 \\ 0 & 0 & 1 & -i \end{pmatrix}$ is the unitary transformation matrix, and, M_+ and M_-, respectively, refer to the pseudospin-up and pseudospin-down matrices, which have the expressions of

$$M_\pm = \begin{pmatrix} \omega_d^2 & \frac{1}{2}[(a + a^*) q_x \pm i(b^* - b) q_y] \\ \frac{1}{2}[(a + a^*) q_x \pm i(b - b^*) q_y] & \omega_p^2 \end{pmatrix}. \tag{30}$$

And N is the coupling matrix,

$$N = \begin{pmatrix} 0 & \frac{1}{2}[(a - a^*) q_x + i(b + b^*) q_y] \\ \frac{1}{2}[(a^* - a) q_x + i(b^* + b) q_y] & 0 \end{pmatrix}. \tag{31}$$

The physics described by Supplementary Equation (29) resembles that of the quantum spin Hall Effect in the electronic systems. The parity inversion shown in Fig. 5a leads to the formation of the acoustic quantum spin Hall insulator. The sign of the Dirac mass

is determined by the frequency difference between the d-wave and the p-wave states, i.e., $\omega_d - \omega_p$.

Supplementary Note 13: Topological index for the first band gap

As depicted in Fig. 5 of the main text, the topological transition in the first band gap can be characterized by the parity inversion at the M point, owing to the fact that identifying centrosymmetric systems with inverted parity eigenvalues is one of the most successful strategies for finding topological materials. Here, to better understand the topological property of the first band gap, we further calculate its overall topological index. According to Supplementary reference [4], for crystalline insulators with C_n symmetry, the band topology can be determined by comparing the rotation representations of the occupied bands at the high symmetry points (HSPs) in the first Brillouin zone (BZ).

Our SC respects C_2 symmetry regardless of the rotation angle θ, whose HSPs include Γ, X, Y and M points, as shown in the inset in Fig. 5b. At these points, the Hamiltonian commutes with C_2 operator, and hence, the wavefunctions at these points are also the eigenfunctions of the C_2 operator. This allows us to use the C_2 eigenvalues at the HSPs as labels for the rotation representations. The eigenvalues of C_2 operator at an HSP Π are denoted as $\Pi_p = e^{2\pi i(p-1)/2}$, with $p = 1, 2$ corresponding to different rotation representations. The integer topological invariant is defined as in Supplementary reference [4]

$$[\Pi_p] = \#\Pi_p - \#\Gamma_p, \tag{32}$$

where Π represents X, Y and M, $\#\Pi_p$ and $\#\Gamma_p$ are the number of bands below the band gap with eigenvalues Π_p and Γ_p, respectively. Intuitively, six integers can be derived based on the above formula. However, since the total number of bands below the gap is constant over the entire BZ, we have

$$[X_1] + [X_2] = [Y_1] + [Y_2] = [M_1] + [M_2], \tag{33}$$

which indicates that three of them are redundant and the topological index representation can be reduced to

$$\chi = ([X_1], [Y_1], [M_1]). \tag{34}$$

In our case, the C_2 rotation eigenvalues (i.e., parity) of the first two bands are always (1, 1) at the Γ point, (1, -1) at the X and Y points, regardless of the rotation angle θ. However, the C_2 rotation eigenvalues at the M point flip signs when θ crosses 90°, as shown in Fig. 5a of the main text. When θ belongs to the region $(0°, 90°)$, the C_2 rotation eigenvalues at the M point equal to 1 for the first two bands, and the above

topological index can be directly obtained as $\chi = (-1,-1,0)$. In contrast, when θ belongs to the region $(90°, 180°)$, the C_2 rotation eigenvalues at the M point are -1 for the first two bands. The topological index is then $\chi = (-1,-1,-2)$. These analyses indicate that the two gapped phases, $\theta \in (0°, 90°)$ and $\theta \in (90°, 180°)$, belong to distinct topological phases that cannot be deformed into one another without closing the first band gap when the symmetry is preserved. The above theory is equivalent to and consistent with the theoretical descriptions in the main text where the identification of topological phase transition is based on the parity inversion at the M point.

Supplementary Note 14: Glide symmetry for the edges in Figure 5 and its physical consequence

In the main text, Fig. 5a presents the evolution of parities for the eigen-states (below and above the band gap) at the M point as functions of the rotation angle, which indicates a parity inversion and characterizes the topological transition in the framework of topological crystalline insulators. Such a sign-change is associated with the bulk gap closing-reopening (see Fig. 5b in the main text). In the bulk band gap, there exist a pair of gapless pseudospin-dependent edge states. Here, we remark that the gapless nature of these edge states is guaranteed by the glide symmetries on the edges, which are preserved when the arch-shaped objects consisting the supercell have opposite rotation angles, i.e., $\theta_{\mathrm{OI}} = -\theta_{\mathrm{TI}}$ (up to an integer of $180°$). In the case shown in Fig. 5, $\theta_{\mathrm{OI}} = 45°$ and $\theta_{\mathrm{TI}} = 135°$. It is observed from Supplementary Figure 20 that this edge configuration also has a glide symmetry $G_{\mathrm{edge}} \coloneqq (x, y) \rightarrow (-x, \frac{a}{2} + y)$. With such a symmetry, we can construct an anti-unitary operator $\Theta_{\mathrm{edge}} = G_{\mathrm{edge}} * \mathcal{T}$ ($\mathcal{T}$ is time-reversal operator, i.e., complex conjugation). Note that at $k_y a = \pi$ the operator Θ_{edge} is an invariant operator, and $\Theta_{\mathrm{edge}}^2 = -1$. Therefore, the edge states have double degeneracy at the $k_y a = \pi$ point where the pseudo-spin up and pseudo-spin down branches intersect with each other. Such Kramers-like degeneracy ensures that the helical edge states are gapless. This is protected by the glide symmetries on the edges.

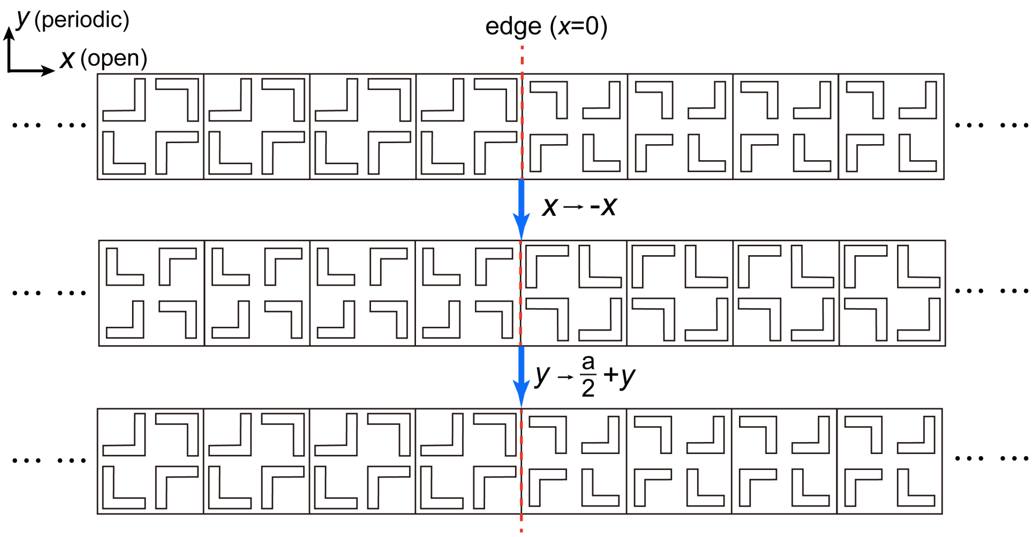

Supplementary Figure 20 | Illustration of the glide symmetry for the *y*-edge when a $45°/135°$ supercell is considered. The structure of the supercell is invariant under the glide transformation (denoted as $G_{\rm edge}$), as illustrated here.

Supplementary Note 15: Robustness of the gapless edge states in the first band gap

In this section, we perform numerical investigation on the robustness of the gapless edge states in the first band gap (i.e., the band gap mimicking the quantum spin Hall effect). Similar to the simulations in Note 7, here we consider the excitations of the edge states when two types of defects, symmetry-preserving defects and symmetry-breaking defects, are introduced into the system. The defect-free structure consists of a SC of 5×20 unit cells with $\theta = 45^{\circ}$, terminated by a SC of the same size with $\theta = 135^{\circ}$. The simulated results are presented in Supplementary Figure 21. Because the gapless nature of the edge states is protected by the glide symmetry on the edge, the "amplification" of the structure in some unit-cells may break the glide symmetry on the edge, unless all the boundary unit-cells connected by the glide reflection have such amplifications. For simplicity, we add four cylinders at the center of each quarter of the unit-cell for all unit-cells along two rows parallel to the boundary, which are connected by the glide reflection. This type of defects preserves the edge glide symmetry. We perform such deformations to the supercell as illustrated in Supplementary Figure 21. The simulation (shown also in Supplementary Figure 21) indicates that the edge spectrum changes little when such symmetry-preserving defects are introduced. In contrast, the amplification-type defects break the glide symmetry on the edge and lead to considerably modified transmission spectrum in the edge-probe.

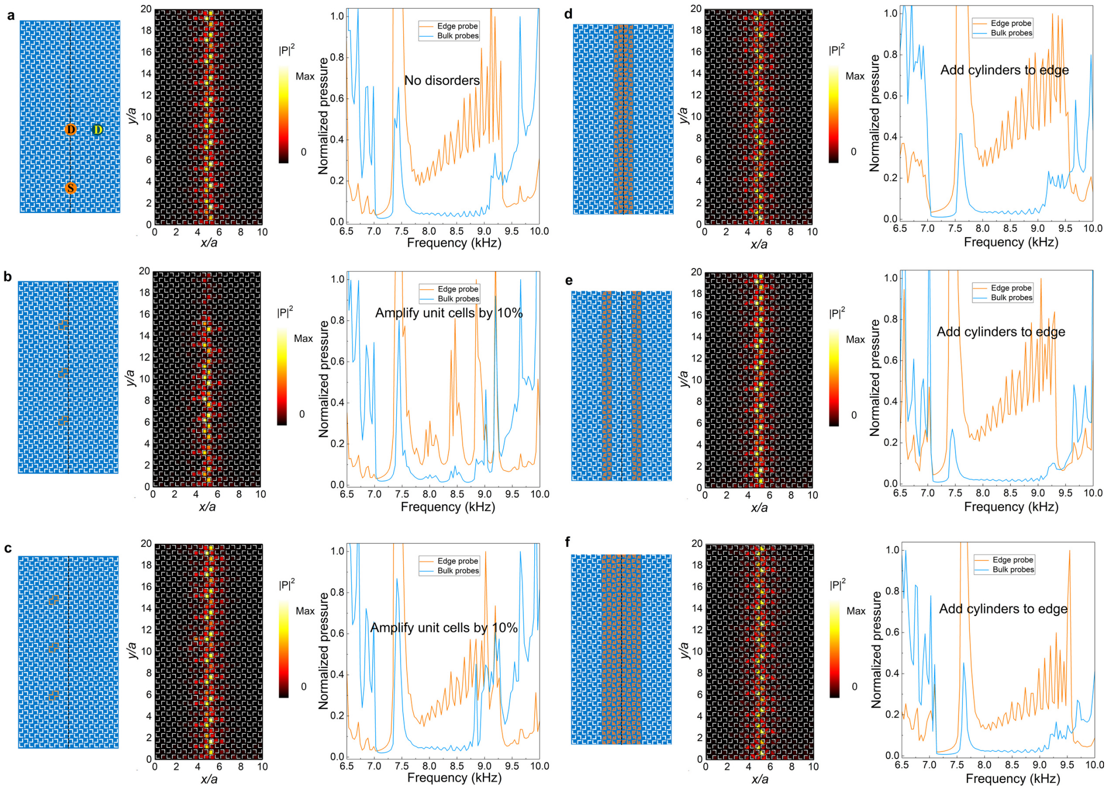

Supplementary Figure 21 | Simulation on the robustness of the edge states in the first band gap. For each supercell structure with or without defects. The left panel schematically illustrates the defects, the middle panel illustrates the edge state filed pattern, the right panel gives the transmission spectrum. The configuration of the source and detector in the pump-probe calculation is illustrated in **a**, including both bulk and edge probes. **a**, No disorder. **b-c**, Symmetry-preserving disorders. **d-f**, Symmetry-breaking disorders.

Supplementary Note 16: The helical edge states and pseudospin-dependent edge state propagation

In Fig. 5 of the main text, we have presented the edge states induced by the dipole band gap (the first band gap), which are shown to mimic the helical edge states in the quantum spin Hall insulators. To visualize the pseudospin-dependent edge state propagation, we present in Supplementary Figure 22 the simulations on the excitations of the edge states by point-like sources carrying negative (the left panel) and positive (the right panel) orbital angular momenta. The point-like source is made of four closely located point sources which have a phase winding of $\pm 2\pi$. It is clearly observed that the acoustic waves travel upwards in the former case and travel downwards in the latter case, with no visible backscattering. These observations are consistent with the analyses in Fig. 5d in the main text where the same pseudo-spin-wavevector locking feature for the helical edge states as that in quantum spin Hall insulators is found.

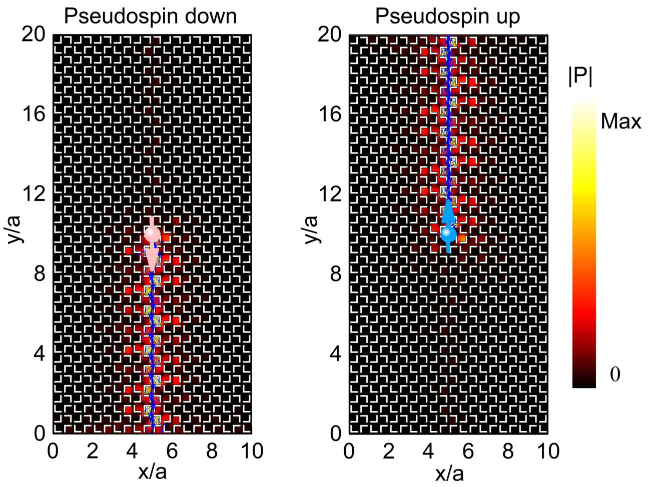

Supplementary Figure 22 | Pseudospin dependent propagation of the helical edge states in the first band gap. Pseudospin dependent unidirectional propagation of the helical edge states (studied in Fig. 5 in the main text) in the lowest acoustic band gap, as excited by point-like sources (the light-pink and light-blue spheres) carrying negative (left) and positive (right) orbital angular momenta. Note that the energy flow ("Poynting vectors") of the propagating edge states are marked by small blue arrows. The light-pink and light-blue arrows highlight the propagation directions of the pseudo-spin polarized edge states.

Supplementary References

[1] Benalcazar, W. A., Bernevig, B. A. & Hughes, T. L. Quantized electric multipole insulators. *Science* **357**, 61-66 (2017).

[2] Benalcazar, W. A., Bernevig, B. A. & Hughes, T. L. Electric multipole moments, topological multipole moment pumping, and chiral hinge states in crystalline insulators. *Phys. Rev. B* **96**, 245115 (2017).

[3] Liu, F. & Wakabayashi, K. Novel topological phase with zero Berry curvature. *Phys. Rev. Lett.* **118**, 076803 (2017).

[4] Benalcazar, W. A., Li, T. H., & Hughes, T. L, Quantization of fractional corner charge in C_n-symmetric higher-order topological crystalline insulators. *Phys. Rev. B* **99**, 245151 (2019).
